# Phytochelatins Bind Zn(II) with Micro- to Picomolar Affinities without the Formation of Binuclear Complexes, Exhibiting Zinc Buffering and Muffling Rather than Storing Functions

**DOI:** 10.1021/acs.inorgchem.4c01707

**Published:** 2024-06-07

**Authors:** Marek Łuczkowski, Weronika Leszczyńska, Joanna Wątły, Stephan Clemens, Artur Krężel

**Affiliations:** ‡ Department of Chemical Biology, Faculty of Biotechnology, University of Wrocław, Joliot-Curie 14a, 50-383 Wrocław, Poland; § Department of Plant Physiology, Faculty of Biology, Chemistry and Earth Sciences, University of Bayreuth, 95440 Bayreuth, Germany

## Abstract

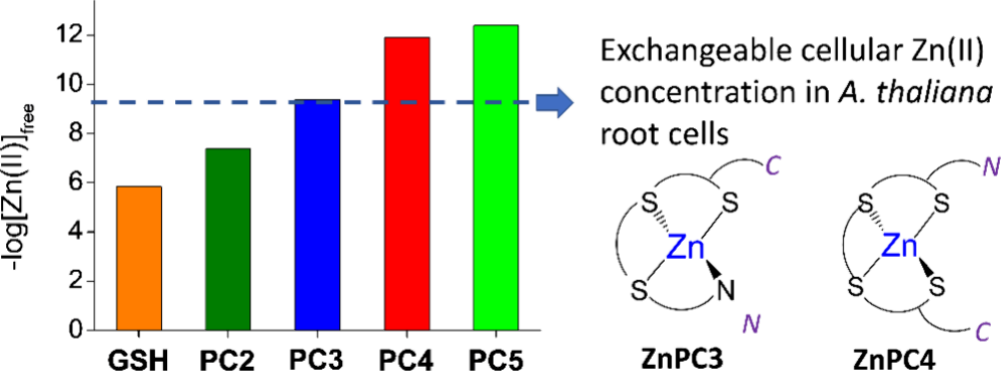

Phytochelatins (PCs) are poly-Cys peptides containing a repeating γ-Glu-Cys motif synthesized in plants, algae, certain fungi, and worms by PC synthase from reduced glutathione. It has been shown that an excess of toxic metal ions induces their biosynthesis and that they are responsible for the detoxification process. Little is known about their participation in essential metal binding under nontoxic, basal conditions under which PC synthase is active. This study presents spectroscopic and thermodynamic interactions with the PC2–PC5 series, mainly focusing on the relations between Zn(II) complex stability and cellular Zn(II) availability. The investigations employed mass spectrometry, UV–vis spectroscopy, potentiometry, competition assays with zinc probes, and isothermal titration calorimetry (ITC). All peptides form ZnL complexes, while ZnL_2_ was found only for PC2, containing two to four sulfur donors in the coordination sphere. Binuclear species typical of Cd(II)-PC complexes are not formed in the case of Zn(II). Results demonstrate that the affinity for Zn(II) increases linearly from PC2 to PC4, ranging from micro- to low-picomolar. Further elongation does not significantly increase the stability. Stability elevation is driven mainly by entropic factors related to the chelate effect and conformational restriction rather than enthalpic factors related to the increasing number of sulfur donors. The affinity of the investigated PCs falls within the range of exchangeable Zn(II) concentrations (hundreds of pM) observed in plants, supporting for the first time a role of PCs both in buffering and in muffling cytosolic Zn(II) concentrations under normal conditions, not exposed to zinc excess, where short PCs have been identified in numerous studies. Furthermore, we found that Cd(II)-PC complexes demonstrate significantly higher metal capacities due to the formation of polynuclear species, which are lacking for Zn(II), supporting the role of PCs in Cd(II) storage (detoxification) and Zn(II) buffering and muffling. Our results on phytochelatins’ coordination chemistry and thermodynamics are important for zinc biology and understanding the molecular basis of cadmium toxicity, leaving room for future studies.

## Abstract 2

Phytochelatins have been known for decades as a major acute response mechanism in plants to growth-inhibiting concentrations of toxic metal ions. Recent interest focuses on their role in the administration of metallic nutrients like Zn(II) under growth-supporting conditions. Comprehensive analysis of Zn(II) binding capacities and complex stoichiometries addresses plant physiology to highlight the role of phytochelatins (PCs) unambiguously in Zn(II) buffering, muffling, and trafficking, rather than preclusion-oriented storage in plants.

## Introduction

Zinc, alongside iron, ranks as one of the most abundant micronutrients in the biosphere. On average, approximately 8–10% of eukaryotic proteins incorporate at least one Zn(II) ion as a cofactor or structure-determining moiety.^1,2^ To fully appreciate its beneficial chemical properties, the cellular exchangeable Zn(II) concentration must be tightly regulated to prevent toxic effects and damage to cellular structures.^3,4^ The stabilities of complexes formed by first-row transition metals follow the principles of the Irving-Williams series (Zn(II) < Cu(II) > Ni(II) > Co(II) > Fe(II) > Mn(II)).^5^ Consequently, Zn(II), with its relatively high affinities toward S-, N-, and O-based ligands, could impact the function of other metalloproteins and displace metal ions ranked downstream in the series. Therefore, cellular systems have evolved several mechanisms to maintain the pool of exchangeable Zn(II) within the range of high picomolar to low nanomolar concentrations in the cell plasma.^6^ Plants share similar mechanisms with other eukaryotes to maintain cellular metal ion homeostasis.^7^ This involves collaborating systems fundamental for the maintenance of cellular metal ion homeostasis, including ion transporter proteins, storage and regulatory/buffering proteins such as metallothioneins (MTs), and low-molecular-weight ligands (LMWLs) forming stable complexes with transition metal ions.^8−10^ The first line of regulation relies on LMWLs that either sequester metal ions in the soil or bind them right after they enter the epidermal cells of the root.^8^ LMWLs can be categorized based on their chemical nature and tissue location. Root cortical and epidermal cells, as well as those surrounding the xylem, are the most active sites for the synthesis and secretion of small molecule metal binding ligands.^8,11^ Several key metabolites such as citrate, malate, ATP, and amino acids like histidine or glutathione (GSH) are certainly involved.^8^ The repertoire of potential small molecule metallo-interactors is further supplemented by specialized metabolites such as *S*-adenosylmethionine derivatives (e.g., nicotianamine and deoxymugineic acid), phytin, coumarins, and GSH derivatives known as phytochelatins (PCs).^8,9,12^ However, knowledge regarding their cellular and tissue distributions remains limited at best. Most of these molecules exert their metal binding preferences intracellularly by chelating exchangeable Zn(II) to form a subnanomolar zinc pool in cell plasma, with levels approximately ∼400 pM in *Arabidopsis thaliana* roots.^15^ Determining and quantifying complex species *in vivo* have been a challenge for over 50 years, and so far, no clear picture has emerged.

**Scheme 1:**
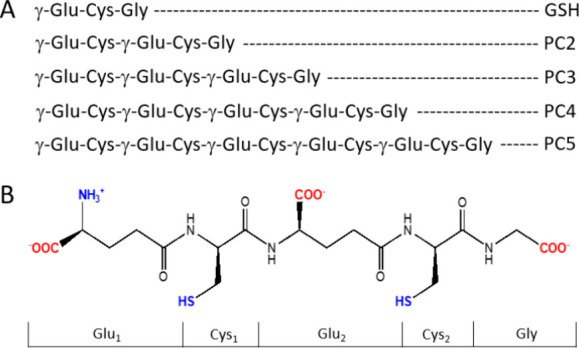
Sequences of γ-Glu-Cys-Containing Peptides Investigated in This Study (A) Primary structure of GSH and PC2-PC5; (B) exemplary structure of PC2. Blue and red colors indicate groups of basic and acidic character, respectively. N-terminal amine group is protonated as in acidic pH used in spectroscopic and potentiometric studies.

Thiol-based LMWLs such as cysteine, glutathione, and phytochelatins (PCs) are more abundant in the reductive environment of the cell plasma. Sulfur donors undeniably exhibit the highest affinity for soft and borderline metal ions among all potential donors. PCs consist of repeating γ-Glu-Cys segments in their sequence, typically presented as (γ-Glu-Cys)_*n*_-Gly.^16^ Depending on their length, they are named PC2, PC3, PC4, etc., where *n* equals 2, 3, and 4, respectively (Scheme 1). They are synthesized by a cytosolic PC synthase (PCS), which in most plant species is activated in the presence of metal excess, leading to substantial fluctuations in PC concentration, sometimes reaching millimolar levels; however, their production is limited by the concentration of GSH and its biosynthesis.^17−19^ PCs are well-known for sequestering heavy metal ions for transfer and storage in the vacuole.^19−21^ An increase in their concentration has been observed in response to a range of metal ions, including Cd(II), Pb(II), Zn(II), Sb(III), Ag(I), Ni(II), Hg(II), Cu(II), Sn(II), Au(I), Bi(III), Pt(II), Rh(III), Pd(II), AsO_4_^3–^, and SO_3_^2–^.^21−23^ The involvement of the PC-dependent tolerance system in the maintenance of Cu(I) and Zn(II) homeostasis in plants is somewhat controversial, as evidence has been presented both for and against the role of PCs in these phenomena.^24,25^ The majority of studies supporting the role of PCs were conducted at metal ion concentrations far beyond those that induce minimal growth inhibition. However, there is a basal rate of PC synthesis even in the absence of metal excess.^17^ Therefore, PC formation occurs continuously and is not just a phenomenon observed under unrealistic metal exposure conditions. The Zn(II) buffering role of PCs under natural growth-supportive conditions, when only this basal PC synthesis takes place, remains to be elucidated.

While phytochelatins are widespread in the plant (not only) kingdom, relatively little information regarding their metal-binding properties has been obtained thus far. Various analytical techniques including chromatographic methods (SEC, HPLC), electrophoretic techniques (CZE), tandem approaches with electrospray ionization-mass spectrometry (ESI-MS), and inductively coupled plasma mass spectrometry (ICP-MS, SEC-ICP-MS) have been employed to quantify and study the formation of complexes between glutathione (GSH) and PCs with nonessential toxic metal ions.^26−30^ ESI-MS has also been utilized for the characterization of Cd(II) complexes from standard mixtures injected directly into the ESI source.^31−33^ Other approaches such as potentiometry, UV–vis spectroscopy,^34−36^ circular dichroism (CD) spectroscopy,^37^ proton nuclear magnetic resonance (^1^H NMR) spectroscopy,^34,38,39^ extended X-ray absorption fine structure (EXAFS) spectroscopy,^40,41^ isothermal titration calorimetry (ITC), and voltammetry/polarography were mostly limited to characterization of toxic metal ions complexes.^42−44^ Investigations aimed to evaluate the physicochemical aspects of Zn(II) interactions of Zn(II) with PCs have been limited to a few articles, primarily focusing on PC2–PC4 peptides. Spectroscopic studies (UV–vis and ^1^H NMR) have reported that PC2 forms ZnPC2 complexes with different stoichiometry compared to Cd(II), which may form polynuclear complexes.^37,39^ Application of ITC and voltammetry for PC3 and PC4 systems indicated a 1:1 complex stoichiometry with binding constants of ∼10^5^ M^–1^.^42,43^ ESI-MS studies for PC4 and PC3 indicated the capability of these peptides to form ternary Zn(II)/Cd(II) complexes.^43,44^

In this study, we present interactions of PCs with essential Zn(II) from various physicochemical and thermodynamic perspectives using the PC2–PC5 peptide series (Scheme 1). The investigations employ mass spectrometry, UV–vis spectroscopy, potentiometry, competition assays with zinc probes (Zincon and PAR), and ITC calorimetry. Our primary objective is to determine the stoichiometry of formed Zn(II)-PC complexes and their stability using reliable methods. A specific focus lies in elucidating the molecular foundations of the complex’s formation and understanding the reasons behind the differences in stability constants among the various PC peptides. We aimed to define the molecular basis behind the arbitrary numbers for understanding interactions between Zn(II) and PCs under physiological conditions in plants and certain animal species where these peptides are present under normal, nontoxic conditions.

## Experimental Section

### Materials

The following reagents were procured from Sigma-Aldrich: 1,2-ethanedithiol (EDT), thioanisole, anisole, triisopropylsilane (TIPS), and Fmoc-Glu(OH)-OtBu. The metal-chelating resin Chelex 100 was obtained from Bio-Rad. Sodium perchlorate was purchased from Acros Organics. Tris(hydroxymethyl)aminomethane (Tris base) was sourced from ROTH, while 4-(2-hydroxyethyl)-1-piperazineethanesulfonic acid (HEPES) was acquired from BioShop. *N*,*N*-Dimethylformamide (DMF) and acetonitrile (MeCN) were procured from VWR Chemicals. NaCl, NH_4_HCO_3_, acetic anhydride, diethyl ether, dichloromethane (DCM), and dimethyl sulfoxide (DMSO) were obtained from Avantor Performance Materials Poland (Gliwice, Poland). Tris(2-carboxyethyl)phosphine hydrochloride (TCEP), 1-methyl-2-pyrrolidinone (NMP), *N*,*N*,*N*′,*N*′-tetramethyl-*O*-(1*H*-benzotriazol-1-yl)uronium hexafluorophosphate (HBTU), trifluoroacetic acid (TFA), *N*,*N*-diisopropylethylamine (DIEA), piperidine, TentaGel S Ram, and Fmoc-Cys(Trt)-OH were sourced from Iris Biotech GmbH (Marktredwitz, Germany). The concentration of the metal ion salt stock solutions was 0.05 M and was confirmed through a representative series of ICP-MS measurements. All pH buffers underwent treatment with Chelex 100 resin to remove trace metal ion contamination.

### Peptide Synthesis

Phytochelatins (PC2-PC5) were synthesized via solid-phase synthesis on Fmoc-Gly preloaded Wang resin (0.68 mmol/g substitution) using the Fmoc strategy either manually or with the Activo P-11 peptide synthesizer (Activotec). Glutamic acid with a γ-peptide bond was introduced using commercially available Fmoc-Glu(OH)-OtBu (Merck), enabling the exclusive formation of a peptide bond with a γ-carboxylate group from the C-terminus. Cleavage and purification were carried out as previously described using a TFA/anisole/thioanisole/EDT/TIPS mixture (88/2/2/5/3, v/v/v/v/v) for 2 h followed by 20% CH_3_COOH/CHCl_3_ extraction (PC2, PC3) or precipitation in cold (−70 °C) diethyl ether (PC4, PC5), respectively.^45,46^ The crude peptide was collected via filtration or centrifugation, redissolved in water, lyophilized, and purified using an HPLC system (Waters 2487 or Varian Prostar) on a Phenomenex C18 or Varian Pursuit XRs C18 column employing a gradient of MeCN in 0.1% TFA/water from 0% to 40% over 20 min (Phenomenex column) and 0% to 100% over 45 min (Varian column). Purified peptides were identified using an API 2000 ESI-MS spectrometer (Applied Biosystems). The identified peptides and calculated average masses are provided in Table S1.

### UV–vis Spectroscopy

UV–vis spectra of Zn(II)-PC systems were recorded using a Jasco V-650 spectrophotometer (JASCO) at 25 °C in a 1 cm quartz cuvette across the range of 200–280 nm.^34,47^ Two spectra were acquired and then averaged. Spectroscopic titrations of 10 μM peptides (PC2–PC5) were conducted in chelexed 20 mM Tris·HCl buffer (100 mM NaClO_4_, pH 7.4) with 5 mM ZnSO_4_ to achieve a final Zn(II)-to-peptide molar ratio of up to 2.0. TCEP was added at a 4–5 molar excess over each cysteine residue as a weakly metal-binding disulfide reducing agent with log *K*_7.4_^ZnL^ = 2.5, and all titrations were carried out under an argon atmosphere.^48^ After the addition of each portion of the ZnSO_4_ stock solution, all samples were equilibrated for 2 min. The pH titration of Zn(II)-GSH and Zn-PC systems was conducted within the range of 4 to 9, with the formation of the complex monitored at 220 nm. To achieve this, 10 μM peptide solutions containing 10 μM ZnSO_4_ were prepared in 0.1 M NaClO_4_, acidified to approximately pH 4, and rapidly titrated with 0.1 M NaOH. All Co(II) complexation studies were carried out in an oxygen-free atmosphere inside a glovebox due to the extreme air sensitivity of Co(II) complexes. Spectroscopic titrations of 250 μM peptides (PC2–PC5) with 50 mM Co(NO_3_)_2_ were performed in 20 mM TES buffer at pH 7.4 (*I* = 0.1 M from NaF).^49^ No TCEP was used in these studies. UV–vis spectra were recorded using a Jasco V-750 spectrophotometer (JASCO) at 25 °C in a 1 cm quartz cuvette spanning the range of 220–900 nm.

### Mass Spectrometry

The binding of Zn(II) to peptides PC2–PC5 and their stoichiometry were monitored in a series of samples with various metal-to-peptide ratios using ESI-MS experiments conducted on an amaZon SL ion trap (IT) mass spectrometer (Bruker Daltonik GmbH, Bremen, Germany) in both positive-ion and negative-ion modes. Peptides were dissolved in 10 mM NH_4_HCO_3_ (pH ∼ 8) to final concentrations of 50 to 100 μM depending on the Zn(II):PC ratio for PC2–PC5. Spectra were recorded for metal-free peptides and their mixtures with Zn(CH_3_COO)_2_ at metal-to-peptide ratios of 0.5:1, 1:1, and 2:1.^50,51^ Source parameters were set as follows: sample flow at 3 μL/min, ion source temperature at 200 °C, and nitrogen flow at 5 L/min with a pressure of 8 psi. Spectra were scanned within the *m*/*z* 100–2200 range. The system was calibrated in positive-ion mode using an ESI-L tuning mix (Agilent Technologies, Santa Clara, California, USA) before data acquisition. Monoisotopic masses, *m*/*z* values, and fragment ion structures were calculated and interpreted by using the Compass DataAnalysis 4.0 program (Bruker Daltonik) software.

### Potentiometric Titration

The protonation constants of the PCs and the stability constants of their Zn(II) complexes in the presence of 4 mM HNO_3_ and 96 mM KNO_3_ (*I* = 0.1 M) were determined at 25 °C under an argon atmosphere using pH-metric titrations over a range of 2.5–10.8 (Molspin automatic titrator, Molspin), employing standardized 0.1 M NaOH as a titrant. The concentration of NaOH was accurately determined by titrating a 4.0 mM standard solution of potassium hydrogen phthalate prepared immediately before the measurement. Changes in pH were monitored using a combined glass–Ag/AgCl electrode (Biotrode, Methrom), calibrated daily in hydrogen concentrations using 4 mM HNO_3_ (*I* = 0.1 M).^52^ Sample volumes ranging from 1.7 to 2.0 mL, ligand concentrations of 0.5–1.0 mM, and Zn(II)-to-ligand ratios of 0.5:1 to 1:1 were employed. Data analysis was performed using the Hyperquad program.^53^ The ionic product of the water used in data processing was determined to be 13.80, corresponding to a 0.1 M ionic strength.^52^

### Isothermal Titration Calorimetry (ITC)

The binding of Zn(II) to PC peptides was monitored using a NanoITC calorimeter (TA Instruments, USA) at 25 °C with a cell volume of 1 mL. All experiments were conducted in HEPES buffer (ionic strength of 0.1 M from NaCl) at pH 7.4, with 600 μM TCEP employed as a nonmetal-binding reducing agent.^47,48^ The concentration of PC peptides (titrate) was set at 50 μM, while the ZnSO_4_ (titrant) concentration was maintained at 0.5 mM. Following temperature equilibration, successive injections of the titrant were introduced into the reaction cell, each increment being 5.22 μL at 300 s intervals, with stirring at 250 rpm. Control experiments to determine the heats of titrant dilution were conducted using identical injections in the absence of titrate. The net reaction heat was derived by subtracting the heat of dilution from the corresponding total heat of reaction. Data preprocessing was carried out using NanoAnalyze software (version 3.12.5) for the Nano-ITC calorimeter.

## Results

### Zn(II)-PC Interactions Monitored by ESI-MS

Mass spectrometry was employed to verify the identity and purity of the investigated thiol peptides, as outlined in Table S1, and to monitor the metal binding stoichiometry of the PC series.^54,55^ It is important to note that the qualitative analysis was conducted in the gas phase, providing limited information about equilibria in solution. Nevertheless, this information should not be extrapolated into a quantitative model, as the ratios of detected species, influenced by their disparate ionization coupled with dissociation of species, vary during the phase transition.^55^ For this reason, irrespective of the molar Zn(II)-load of the ligand, only equimolar complex species were identified, as biscomplexes are typically unstable under experimental conditions.^56^ Binuclear species were detected for only the longest studied homologue. Signals and isotopic distributions in the experimental and simulated spectra are in perfect agreement, supporting the accurate interpretation of species formed in (NH_4_)_2_CO_3_, corresponding to ionization at pH ∼ 8.^37^

In the Zn(II)-PC2 system (Figure S1), the most intense signals correspond to the [L]^+^ species (*m*/*z* value at 540.3) and its [Zn+L]^+^ complex species (*m*/*z* value at 602.2), along with its chloride adduct [Zn+L+Cl]^+^ (*m*/*z* value at 640.1). PC3 presents a similar pattern, although both single and doubly charged complex signals (834.2 *m*/*z*, *z* = 1 [L+Zn]^+^ and 417.7 *m*/*z*, *z* = 2), in addition to ligand ions (772.3 *m*/*z*, *z* = 1 and 386.7 *m*/*z*, *z* = 2) with no chloride adduct, are detected (Figure S2). PC4 (1004.3 *m*/*z*, *z* = 1; 502.7 *m*/*z*, *z* = 2), providing four thiolate binding sites, preferably forms equimolar complexes (533.7 *m*/*z*, *z* = 2) (Figure S3). PC5 (618.8 *m*/*z*, *z* = 2), the only studied homologue capable of forming both equimolar (649.7 *m*/*z*, *z* = 2) and binuclear species (682.7 *m*/*z*, *z* = 2), exhibits the latter regardless of the applied Zn(II)-load at both 1:1 and 1:2 molar ratios (Figure S4).

Mass spectrometry analysis indicates that, unlike Cd(II), Zn(II) complexation primarily yields equimolar species.^37^ Although, due to previously mentioned ionization coupled with dissociation of species, the technique’s limitations preclude the detection of species containing more than one ligand moiety, we are confident they are formed, at least by the shorter PC homologues. Once the metal-to-peptide ratio reaches equality, most homologues of the PC system saturate with sequestered metal ions, aligning with Zn(II)’s coordination preference for the formation of four-coordinate species. PC5, with its five γ-Glu-Cys segments, can feasibly bind additional Zn(II) ions. While such species are detected through MS analysis, their molecular structure and atomic composition are challenging to predict and may simply result from the experimental conditions during phase transition.

### Complexation at Constant pH Monitored by UV–vis Spectroscopic Titrations

To the best of our knowledge, there is a limited number of physicochemical reports on the Zn(II)-phytochelatin system, especially focusing on the longer PC homologues (refer to the Introduction). Consequently, we conducted systematic spectroscopic studies on the interactions between Zn(II) ions and the PC2–PC5 series. Initially, we titrated the respective phytochelatins (PC2, PC3, PC4, and PC5) with ZnSO_4_ at a constant pH, and UV–vis spectra were recorded in the range of 200 to 280 nm. These titrations were performed to assess the metal binding properties, particularly the stoichiometry of the formed complexes across the series. Moreover, given that spectroscopic signatures of Zn(II) complexes are relatively poor due to the absence of d–d bands and the presence of only high-energy LMCT (ligand-to-metal charge transfer transitions) bands, we investigated Zn(II)-PC interactions using Co(NO_3_)_2_ in an oxygen-depleted glovebox environment to prevent the oxidation of air-sensitive Co(II)-thiolate complexes. Spectra of the Co(II)-PC systems were recorded across a wide spectral range covering both the LMCT and d–d regions of Co(II) complexes, spanning from 210 to 900 nm. Note that investigation of PC interaction with Zn(II) using CD spectroscopy was impossible due to very low signal intensity, similar to zinc mammalian metallothioneins.^57^

**Figure 1:**
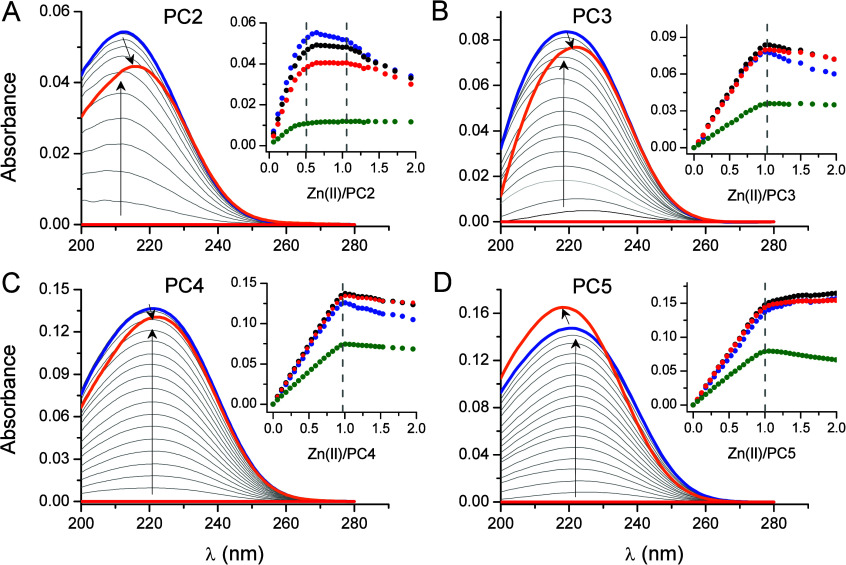
Spectroscopic titrations (differential spectra) of PC2 (A), PC3 (B), PC4 (C), and PC5 (D) (10 μM) with ZnSO_4_ in 20 mM Tris-HCl buffer at pH 7.4 and 25 °C (*I* = 0.1 M from NaClO_4_). Red, blue, and orange spectra denote 0, 1, and 1.5 molar ratio of Zn(II)-to-PC, respectively. Arrows indicate absorbance changes. The insets demonstrate absorbances at particular wavelengths as a function of metal-to-peptide molar ratio. Green, red, black, and blue circles denote absorbances recorded at 240, 225, 220, and 212 nm, respectively.

Commencing with the shortest peptide, the titration of PC2 with ZnSO_4_ results in an increase in absorbance attributed to the S → Zn(II) ligand-to-metal charge transfer band (LMCT), evident in a background increase in the far UV range without characteristic bands (Figure S5A). However, differential spectra (obtained by subtracting the metal-free peptide spectrum from the complex) reveal the emergence of a single band with a maximum at 212 nm (Figure 1A). The maximum absorbance is attained at a Zn(II)-to-PC2 molar ratio of 0.5, indicating the predominant formation of the bis-complex Zn(PC2)_2_ in the solution. The driving force behind its favored formation is the exclusively thiolate binding mode {4S} of the Zn(II) ion, a mode also adopted by numerous Cys-rich proteins such as metallothioneins or zinc finger domains.^3,49,57^ It should be remembered that carboxylates and amine groups also participate in Zn(II) binding in proteins. Indeed, sequential addition of ZnSO_4_ to PC2 results in a red shift of the spectra and constant absorbance up to a Zn(II)-to-PC2 molar ratio of 1.0, beyond which the absorbance at 212 nm begins to decrease. This spectral behavior indicates the formation of equimolar ZnPC2 complex species with a mixed ligand composition, presumably in an {2SNO} or {2S2O} Zn(II) binding environment. The observed red shift in the maximum absorption, corresponding to the energy of the LMCT transition, provides additional support for this interpretation. Further addition of ZnSO_4_ is more likely to result in nonspecific interactions with the peptide.

Spectroscopic data indicate that longer phytochelatin homologues, starting from PC3, form equimolar complex species exclusively (Figure S5B, Figure 1B). Despite PC3 being capable of providing only three sulfur donor atoms, suggesting that the Zn(II) ion may not necessitate a homoleptic tetrathiolate binding environment, no transient bis-complexes or clustered forms were detected. The formation of mixed-ligand species with a {3SN} or {3SO} coordination sphere is the final product of Zn(II) sequestration. A red shift of the maximum absorption band observed for PC3, albeit significantly lower than in the case of PC2, may arise from the unspecific interaction of Zn(II) aliquots added to the saturated complex species or the conformational flexibility of the peptide backbone, where a certain amount of peptide bonds is formed by the γ-carboxylate of Glu and the amine group of the succeeding Cys residue. PC4, providing four thiolate anchors for Zn(II) binding, exclusively forms equimolar species, as indicated by the linear increase in the intensity of LMCT bands in the UV range of spectra (Figure S5C, Figure 1C). Notably, the substantial increase in the extinction coefficient is not accompanied by a shift in the maximum absorption band (minor maximum change) regardless of the presence of an additional γ-Glu-Cys segment. This observation is attributed to the involvement of the fourth thiolate in the coordination sphere of Zn(II). PC5, spanning five γ-Glu-Cys repeats, shares similarities in Zn(II) binding mode with PC4 (Figure S5D, Figure 1D). The observed blue shift after the addition of more than 1 Zn(II) molar equiv may suggest weak binding of the additional Zn(II). PC5, with five thiols, may offer an additional binding site where the metal coordination sphere might be completed by additional peptide donors or even by clustering with the first site. The lack of a characteristic inflection point (inset of Figure 1D) suggests that the second site might be significantly weaker compared to the first one.

**Figure 2:**
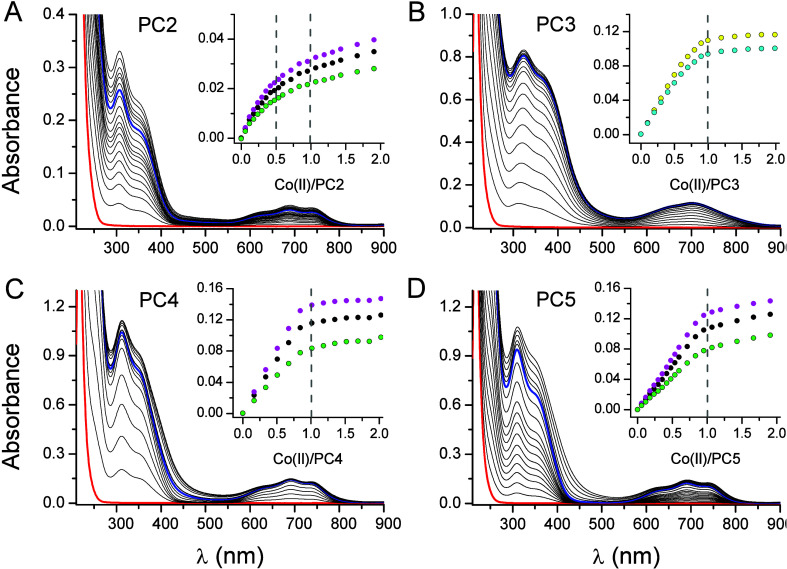
Spectroscopic titrations of PC2 (A), PC3 (B), PC4 (C), and PC5 (D) (250 μM) with Co(NO_3_)_2_ in 20 mM TES buffer at pH 7.4, 25 °C (*I* = 0.1 M from NaF). Red and blue spectra are for 0 and 1 molar ratio of Co(II)-to-PC. The insets demonstrate absorbances at particular wavelengths as a function of metal-to-peptide molar ratio. Green, blue, magenta, yellow, and black absorbances were at 625, 653, 692, 701, and 740 nm, respectively.

**Figure 3:**
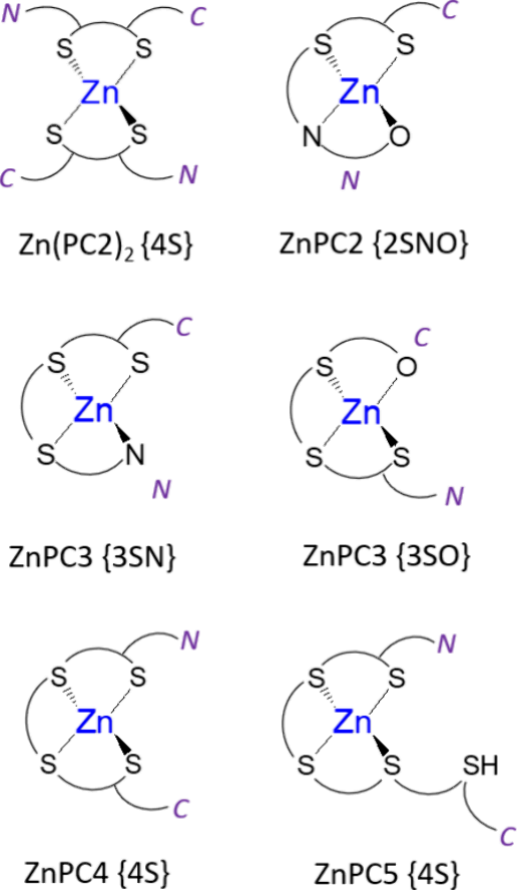
Schematic representation of Zn(II) complexes formed by the series PC2–PC5 at neutral pH with the indication of complex stoichiometry and metal donor composition. N and C denote the N-terminus (γ-Glu residue) and C-terminus (Gly residue) of each PC, respectively.

To elucidate the scientific interpretation of the aforementioned spectroscopic data, the interaction of Zn(II) with PCs was investigated using Co(II), known as the most effective spectroscopic probe for Zn(II) ions to date.^58−60^ The application of Co(II) as a probe provides insights into complex geometry based on the spectroscopic signatures within the d–d range, which are typically inaccessible for Zn(II)-based systems. This envelope is characterized by a low extinction coefficient compared to that of LMCT bands. Therefore, to obtain reasonably good quality recorded spectra, the concentration range of peptides was over 1 order of magnitude higher than that applied in direct Zn(II) titration spectroscopic studies (Figure 2, Figure S6). The titration of PC2 with Co(NO_3_)_2_ indicates the absence of characteristic saturation points (inset of Figure 2A), as observed in the case of ZnSO_4_ titration. The curve’s shape suggests the formation of a much weaker complex than in the case of the Zn(II) counterpart, which is the reason for the lack of observable saturation points under the used conditions. The d–d envelope of the formed complexes is characteristic of a tetrathiolate and tetrahedral coordination environment of the Co(II) ion, evidenced by the energy of three spectral components (630, 692, and 740 nm) and the molar absorption coefficient (Figure S6A).^61^ Although it is challenging to determine due to the lack of signal saturation, it can be estimated to be ∼500–600 M^–1^·cm^–1^. This information strongly suggests that under the utilized conditions only the Co(Cys)_4_ complex is formed, which is only possible when two PC2 molecules form the bis-complex Co(PC2)_2_ (Figure 3).

The titration of PC3 with Co(II) reveals the predominant formation of a complex at a 1:1 Co(II)-to-ligand stoichiometry (Figure 2B). However, an analysis of the d–d envelope reveals that the predominant CoPC3 complex represents a different coordination environment of Co(II) (Figure S6B) from that observed for PC2 biscompelexes. The spectral components of the d–d envelope are more overlapped than in the case of the Co(PC2)_2_ species, suggesting a different, heteroleptic composition of the coordination sphere of the Co(II) ion in the CoPC3 complex, with sulfur donors being dominant, more likely {3SO} or {3SN} (Figure 3). However, the value of the molar absorption coefficient, 460 M^–1^·cm^–1^, and energy of d–d bands, indicates that the complex has a tetrahedral geometry, despite differences in the donors’ composition of Co(II). Titrations of PC4 and PC5 are very similar to each other, indicating the formation of equimolar CoPC4 and CoPC5 complexes (Figure 2C,D). The spectral components of the d–d envelope demonstrate a pattern very similar to that of the Co(PC2)_2_ complex, confirming a {4S} coordination environment of the Co(II) ion (Figure 3, Figure S6C,D). The extinction coefficients of Co(II) complexes of PC4 and PC5 are almost identical, reaching values of 580 and 590 M^–1^·cm^–1^, respectively. Spectroscopic data indicate a low probability of the formation of dimeric or clustered species by longer PC homologues.

### pH-Dependent Formation of Zn(II) Complexes with GSH and PCs

To assess the acidity of PC thiols in the presence of Zn(II) ions, PC peptides were spectrophotometrically titrated (1:1 molar ratio) across a wide pH range, starting from 4, where all thiols are protonated and not bound to Zn(II), up to 9, where thiolates bind to Zn(II). In order to incorporate some acid–base and coordination aspects of reduced glutathione (GSH), this peptide was included in the series since it contains a single γ-Glu-Cys segment. The deprotonation of thiols in the presence of ZnSO_4_ and the simultaneous formation of Zn(II) complexes were monitored at 220 nm, where LMCT bands are formed upon Zn(II) coordination. Experimental points on the isotherm were fitted to Hill’s equation to determine the pH value at which half of the dissociation occurred (inflection point), corresponding to the p*K*_a_′ value, as well as the isotherm slope, which represents the Hill coefficient (*n*). It is important to emphasize that p*K*_a_′ values of thiols determined in such experiments inform about the acid–base properties of the thiol groups within the ligand and also serve as a measure of the particular peptide’s affinity for Zn(II).

**Figure 4:**
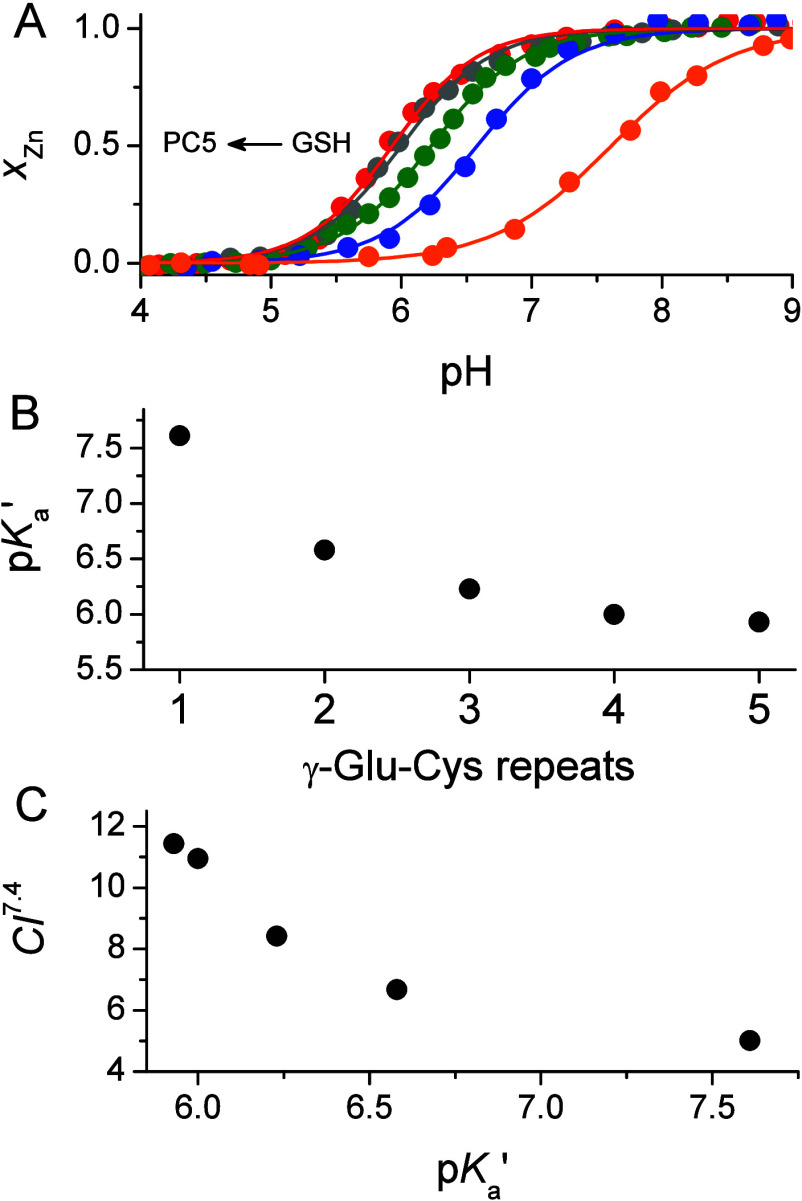
pH-dependent formation of Zn(II) complexes with GSH and PCs. (A) Isotherms of Zn(II) complex formation as a function of pH (metal-to-peptide molar ratio 10 μM:10 μM). Molar fractions (*x*_Zn_) were calculated from the absorbance increase at 220 nm. p*K*_a_′ denotes the inflection point, which corresponds to 50% complex formation. Red, gray, olive, blue, and orange correspond to PC5, PC4, PC3, PC2, and GSH, respectively. (B) Dependence of p*K*_a_′ values on the number of γ-Glu-Cys repeats in GSH and PC2–PC5 series. (C) Relation between CI^7.4^ and p*K*_a_′ values. CI^7.4^ for GSH was calculated based on data from ref (62).

Figure 4A illustrates the isotherms of Zn(II) complex formation as a function of increasing pH. The inflection points (p*K*_a_′) represent the averaged dissociation constants of the thiols under the applied conditions and increase from 5.93 ± 0.02 for PC5 to 6.00 ± 0.02 for PC4, 6.23 ± 0.01 for PC3, and 6.58 ± 0.01 for PC2. The p*K*_a_′ value of GSH is the most basic, equaling 7.61 ± 0.02. The slopes of PC2–PC4 peptide complexation are similar to each other with an average value of 1.3, indicating a lack of high cooperativity in thiol deprotonation. Values above 1.0, however, suggest that Zn(II) binding does not occur fully in a stepwise manner. Hill’s coefficient of GSH was fitted as 0.97 ± 0.03, revealing that thiol deprotonation and thiolate complexation are not dependent on other processes. The comparison of p*K*_a_′ values with the number of γ-Glu-Cys segments in GSH and PC2–PC5 series demonstrates a significant decrease in the values up to four γ-Glu-Cys repeats (Figure 4B). The value for the five segments is almost identical with PC4, indicating high similarities in acid–base properties and Zn(II) affinity. It should be emphasized, however, that the decrease of p*K*_a_′ values is not linear as observed in Cd(II) complexes indicating structural differences in the complexes of both metal ions.^37^

### Stoichiometry and Stability of Zn(II) Complexes Determined Potentiometrically

The acid–base properties of the phytochelatin series have been previously addressed and discussed in our paper detailing their metal binding properties toward Cd(II).^37^ Although there are no significant differences between homologues of the PC series, minor increases in thiol basicity with the increment in the number of γ-Glu-Cys segments are observed based on potentiometric data (Table 1). Of particular interest is how these minor differences translate to metal binding affinities. Potentiometry stands as one of the most precise methods for determining the stability constants of low-molecular-weight peptides due to its sensitivity and the capability to establish a stoichiometric model across a wide pH range. The evaluation of speciation profiles and the assignment of stability constants over this range can lead to more comprehensive and widely appreciated apparent affinity constants. Alternatively, as is often the case in more complex systems, competivity indexes might be calculated.^62^ These values aid in comparing systems of varying affinities at the same pH, offering an overall assessment of the metal affinity of the studied systems and allowing for further discussion of the structural reasons for their differences and the resulting consequences.

**Table 1:** Protonation Constants of PC Peptides Determined Potentiometrically at 25 °C (*I* = 0.1 M from KNO_3_)^a^

	log β_jk_^b^			
species	PC2	PC3	PC4	PC5
HL	10.25 (5)	10.21 (2)	10.20 (3)	10.26 (2)
	*10.25*^c^	*10.21*	*10.20*	*10.26*
H_2_L	19.94 (2)	19.52 (2)	20.14 (2)	20.39 (2)
	*9.69*	*9.31*	*9.94*	*10.13*
H_3_L	28.53 (2)	28.77 (1)	29.49 (3)	29.99 (3)
	8.59	9.25	9.35	9.60
H_4_L	32.83 (3)	37.08 (1)	38.59 (2)	39.32 (2)
	*4.30*	*8.31*	*9.10*	*9.33*
H_5_L	36.00 (2)	41.13 (2)	46.99 (2)	48.30 (2)
	*3.17*	*4.05*	*8.40*	*8.98*
H_6_L	38.43 (5)	44.70 (2)	51.40 (3)	56.68 (1)
	*2.43*	*3.57*	*4.41*	*8.38*
H_7_L		47.48 (2)	55.04 (2)	61.26 (3)
		*2.78*	*3.64*	*4.58*
H_8_L		48.93 (7)	58.47 (3)	65.22 (2)
		*1.45*	*3.43*	*3.96*
H_9_L			61.10(2)	68.75 (3)
			*2.63*	*3.53*
H_10_L			n.d.	72.09 (2)
				*3.34*
H_11_L				74.70 (3)
				*2.61*
H_12_L				76.46 (5)
				*1.76*

a Constants are presented as cumulative log β_jk_ values. Standard deviations of the last digits are given in parentheses. L stands for a peptide (ligand) with acid–base active groups. Numerical values in italics correspond to p*K*_a_ values of the peptides and were derived from cumulative constants. n.d. denotes not detectable under used conditions.

b β(H_j_L_k_) = [H_j_L_k_]/([H]_j_[L]_k_), in which [L] is the concentration of the fully deprotonated peptide.

c log β(H_k_L_j_) – log β(H_k–1_L_j_) = p*K*_a_.

**Figure 5:**
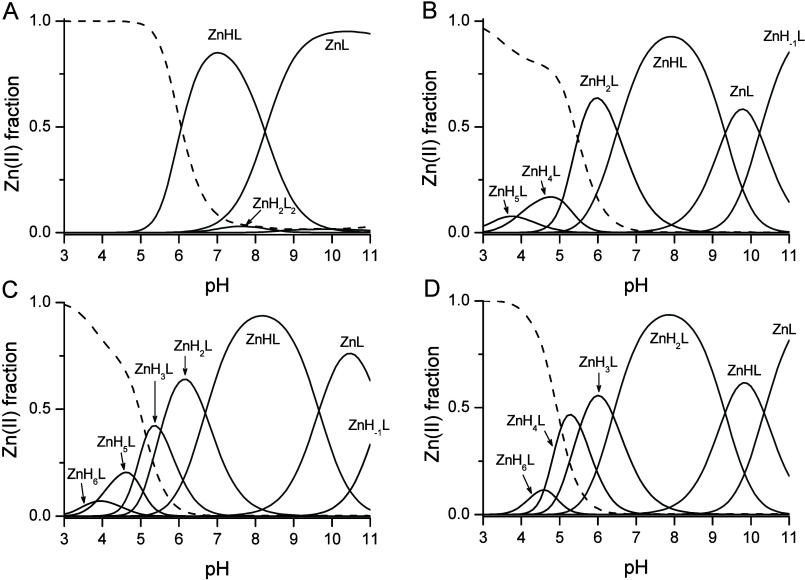
Species distribution profiles for Zn(II) complexes of PC2 (A), PC3 (B), PC4 (C), and PC5 (D) at a 1.0 Zn(II)-to-peptide ratio (500 μM Cd(II) and 500 μM PCs) on the basis of potentiometric results (25 °C, *I* = 0.1 M from KNO_3_). Dashed line corresponds to free Zn(II); solid line corresponds to biscomplexes and mononuclear species, respectively. For clarity, speciations for GSH and PC2 with Zn(II) at ratios 1:1 and 1:2 are presented in Figure S7 and Figure S8 in the Supporting Information.

Stoichiometric models and cumulative stability constants (β_ijk_) of the PC2–PC5 series were determined through potentiometric data analysis using Hyperquad software and are presented in Table 2.^53^ PC2 forms equimolar ZnHL and ZnL, as well as ZnH_2_L_2_ and ZnL_2_ biscomplexes with Zn(II). In the highly alkaline pH range, complex hydrolysis occurs in conjunction with the hydrolysis of hydrated Zn(II) ions. This indicates that depending on the molar ratio between Zn(II) and the ligand PC2 is capable of forming two types of stoichiometries regardless of complex protonation. This characteristic arises from the presence of two thiol groups in one molecule and its tendency to form a homoleptic species. However, at higher Zn(II) concentrations, other atoms or groups from the PC2 molecule take over the role of donors. Figure 5A illustrates the species distribution of Zn(II) complexes at an equimolar ratio, while Figure S7 shows the distribution at twice the excess over the metal. A very similar stoichiometric model is demonstrated by GSH, which also forms equimolar and biscomplex species (Figure S8). The longer homologue, PC3, forms only variably protonated equimolar complexes with the following formulas: ZnH_5_L, ZnH_4_L, ZnH_2_L, ZnHL, ZnL, and ZnH_–1_L (Figure 5B). Unlike PC2, longer PC homologues do not form bis-complex species under predefined experimental conditions where Zn(II) and peptide concentrations are nearly equal. This suggests that PC3, which includes three γ-Glu-Cys segments, will utilize N or O donors (Figure 3), aside from the preferred thiolate sulfurs, to meet the demand of Zn(II) for a pseudotetrahedral binding environment. Zn(II) ion sequestration by PC4 and PC5 is purely tetrathiolate (supported by spectroscopic data) as both provide four thiolates that are perfectly separated in the primary structures (Figure 5C,D). PC4 yields ZnH_6_L, ZnH_5_L, ZnH_3_L, ZnH_2_L, ZnHL, ZnL, and ZnH_–1_L species, while PC5 forms ZnH_6_L, ZnH_4_L, ZnH_3_L, ZnH_2_L, ZnHL, and ZnL complexes. The Zn(II)-promoted increased acidity of the carboxylates and thiols grows with the number of γ-Glu-Cys repeats, as manifested by the initial formation of species with progressively higher numbers of deprotonated functional groups along the PC homological series. For the majority of homologues (PC2, PC3, and PC4), the most predominant species in the neutral pH range is the ZnHL complex, while PC5 sequesters the Zn(II) ion as the ZnH_2_L complex. This suggests that, in the biologically relevant neutral pH range, ZnHL complexes formed by PC2, PC3, and PC4 have the α-amino group remaining protonated, while PC5, in addition, has one of the unbound thiolates protonated, yielding the ZnH_2_L species.

**Table 2:** Zn(II) Stability Constants of PC Peptide Complexes Determined Potentiometrically at 25 °C (*I* = 0.1 M from KNO_3_)^a^

	log β_ijk_^b^			
species	PC2	PC3	PC4	PC5
ZnH_6_L			54.001 (6)	59.630 (8)
ZnH_5_L		43.793 (7)	50.172 (3)	
ZnH_4_L		39.921 (8)		50.443 (2)
ZnH_3_L			40.513 (1)	44.906 (2)
ZnH_2_L		29.798 (2)	34.989 (1)	38.511 (2)
ZnHL	20.020 (2)	23.290 (2)	28.300 (2)	29.186 (3)
ZnL	11.771 (5)	13.963 (3)	18.639 (2)	18.842 (3)
ZnH_–1_L		3.736 (3)	7.363 (3)	
ZnH_2_L_2_	37.34 (1)			
ZnL_2_	20.042 (8)			
ZnH_–1_L_2_	9.340 (9)			

a Constants are presented as cumulative log β_ijk_ values. Standard deviations of the last digits are given in parentheses. L stands for a peptide (ligand) that binds to Zn(II).

b β(M_i_H_j_L_k_) = [M_i_H_j_L_k_]/([M]_i_[H]_j_[L]_k_) in which [L] is the concentration of the fully deprotonated peptide.

**Figure 6:**
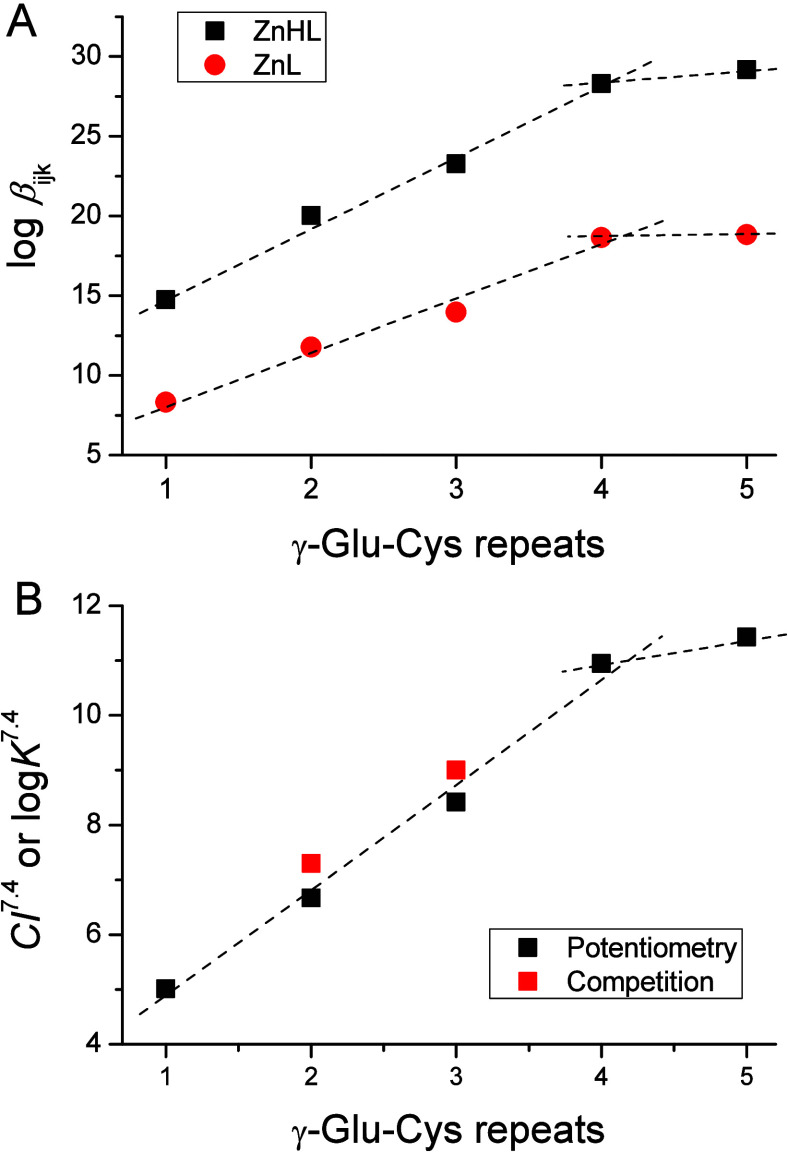
Stability constants of Zn(II)-PC complexes in comparison in the series GSH-PC5. (A) Relation of cumulative constants of ZnHL and ZnL complexes derived from potentiometric titrations. (B) Comparison of the formation constants (log *K*^7.4^) determined in the competition experiments with Zincon or PAR and competivity indexes (CI^7.4^) derived from potentiometric data.

The comparison of cumulative stability constants for physiologically relevant complex species formed by Zn(II) and Cd(II) in the neutral pH range (Table 3) suggests that the stabilities of Zn(II) species are 2 to 3 orders of magnitude weaker than their Cd(II) counterparts. Such a difference arises from the more thiophilic character of Cd(II) ions compared to Zn(II), a phenomenon observed in numerous {4S} systems such as zinc finger and other structural motifs.^47,60,63^ Interestingly, comparing the cumulative constants of ZnHL and ZnL complexes across the series of GSH and PC2–PC4 indicates a linear increase in the stability of both complexes as a function of the γ-Glu-Cys segment, from one for GSH to four for PC4 (Figure 6A). For PC5, the stability constants remain similar to those of PC4. This implies that, regardless of some differences in the acidities of particular acid–base active groups, overall stability linearly depends on the number of sulfur donors per molecule. Further elongation of the peptide does not significantly increase Zn(II) complex stability. A similar tendency of stability constants increase as a function of γ-Glu-Cys repeats was also observed for Cd(II)-PC complexes.^37^

**Table 3:** Comparison of Cumulative Stability Constants (log β_ijk_ Values) Formed by GSH and PCs in Neutral pH Range by Zn(II) and Cd(II)

peptide	major Zn(II) complex species	Zn(II) complex stability constant	respective Cd(II) complex stability constant
GSH	ML	8.31^b^	9.00^c^
	MHL_2_^a^	22.53^b^	24.22^c^
PC2	MHL	20.02	22.82^b^
	MH_2_L_2_^a^	37.34	41.25^b^
PC3	MHL	23.29	26.79^b^
PC4	MHL	28.30	30.63^b^
PC5	MH_2_L	38.51	40.23^b^

a Species present at ligand excess over metal ion.

b Value taken from ref (62).

c Value taken from ref (37).

Although comparing cumulative β_ijk_ constants of Zn(II) complexes of chemically similar peptides is informative, it does not provide information about the affinity at a particular pH and is affected by differences in protonation contents among the studied series of peptides. Better measures of ligand affinities toward metal ions are provided by either apparent formation constants or competivity indexes, which omit the impact of the ligand’s protonation states, although they are valid only for specified conditions. Apparent formation constants can be either determined experimentally at a particular pH by following metal complexation or calculated based on pH-independent constants obtained from potentiometric studies. The competivity index (CI) is more universal as it simplifies all the stoichiometries of metal complexes to an equimolar one and can be easily calculated based on potentiometric data.^62^ To calculate it (eq 1), one needs to first define the Zn(II) complex with the theoretical molecule Z, where [ZnZ] is the sum of all complexes regardless of stoichiometry (Σ_ijk_Zn_i_H_j_L_k_) at a given overall component concentration, while [Z] is Σ_jk_H_j_L_k_ under the applied conditions.
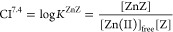
1Here, CI^7.4^ values of the GSH and PC series were calculated for equilibrium reagent concentrations at pH 7.4 and are presented in Table 4. From these values, it is evident that the stability difference between Zn(II) complexes of the longest PC5 (with low picomolar affinity) and the shortest PC2 (with submicromolar affinity) is almost 5 orders of magnitude, which increases to six and a half when GSH (with micromolar affinity) is taken into account. This value translates to approximately −9 kcal/mol, corresponding to approximately −3 kcal/mol of the stabilization Gibbs free energy effect per one γ-Glu-Cys segment from GSH to PC5. Figure 6B illustrates that the CI^7.4^ increase is linear up to PC4 and does not change significantly above the four γ-Glu-Cys repeating segments. This observation is comparable to the comparison of cumulative constants shown in Figure 4A. However, the relationship between CI^7.4^ values and p*K*_a_′ determined in pH-dependent spectroscopic titrations (Figure 4C) is linear only from PC3 to PC5. The p*K*_a_′ for PC2, and especially for GSH, deviates from the linear trend. This indicates that the spectroscopic signal (LMCT band formation) does not correlate with the potentiometric model for GSH and PC2, suggesting the involvement of other donor groups aside from sulfur in Zn(II) complexation in those peptides. It should be noted that this type of relationship remains linear for Cd(II) complexes throughout the entire series from GSH to PC6.^37^

**Table 4:** Competivity Indexes (CI^7.4^) or Apparent Formation (Binding) Constants (log *K*^7.4^) of Zn(II) Complexes with GSH and PC Complexes at pH 7.4^a^

ligand	potentiometry (CI^7.4^)^b^	Zincon or PAR competition (log *K*^7.4^)
GSH	5.01	5.0 ± 0.1
PC2	6.67	7.3 ± 0.1
PC3	8.42	9.0 ± 0.1
PC4	10.95	n.d.
PC5	11.43	n.d.

a CI^7.4^ values were calculated from stability constants determined potentiometrically while log *K*^7.4^ were determined by the competition with Zincon or PAR. n.d. denotes not determined.

b See eq 1.

### Competition Studies of GSH and PCs with Zinc Probes

**Figure 7:**
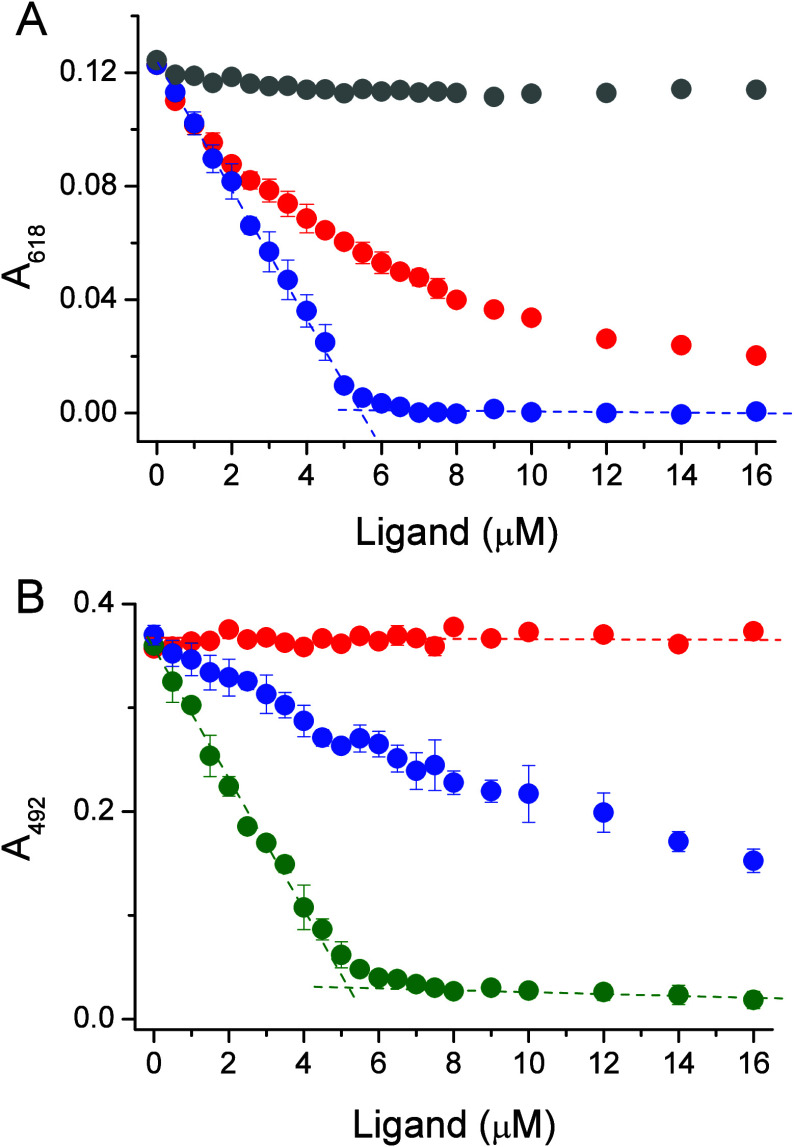
Spectroscopic competition between 100 μM Zincon (A) or PAR (B) with GSH (gray circles), PC2 (red circles), PC3 (blue circles), and PC4 (olive circles). Zincon and PAR were initially saturated with 5 μM ZnSO_4_ in 50 mM HEPES buffer, 100 mM NaCl, 100 μM TCEP, pH 7.4 and then titrated with peptide stocks prepared in 5 mM HCl.

Spectroscopic titrations of the PC series with ZnSO_4_ revealed that all of them (with PC2 being the least) bind Zn(II) with high affinity, although with some differences in stoichiometry. Determination of stability constants from such titrations is either impossible or results in an underestimation of the affinities, as has been shown on several occasions. An alternative to such limitations is the application of competitive ligands that overcome the problems of affinity limitations.^60^ Among all Zn(II)-chelating chromophoric probes, Zincon and PAR are suitable for these competition experiments. The former binds Zn(II) with 1:1 stoichiometry with micromolar affinity (log *K*^7.4^ = 5.68), while the latter forms ZnH_*x*_L_2_ complexes when used in excess. An average formation constant (log *K*_12_^7.4^) of the ZnH_*x*_(PAR)_2_ complex at pH 7.4 is 12.15.^64,65^ Here, both probes were used to compete with the PC series and GSH. For this purpose, 100 μM probe was partially saturated with ZnSO_4_ (5 μM) and then titrated with a stock solution of peptide, and the change in characteristic absorbance of the Zn(II)-probe complex was monitored at 618 and 492 nm in the case of Zincon and PAR, respectively. Figure 7A shows absorbance changes for the competition of GSH, PC2, and PC3 with the ZnZincon complex. It is clear that only PC2 virtually outcompetes the complex, while PC3 is too strong and fully removes Zn(II) from the probe complex. Similarly, PC4 and PC5 form highly stable complexes with Zn(II) and outcompete Zincon. On the other hand, GSH competes very weakly, which is not observable in this range of peptide concentrations (Figure 7A). The same experiment performed at a significantly higher GSH concentration demonstrates clear competition (Figure S9). To calculate formation constants, absorbance values were transformed to the equilibrium concentration of Zn(II)-probe, and the procedure presented by Kocyła et al. was followed.^65^ Determined log *K*^7.4^ constants (Table 4) are comparable with CI^7.4^ calculated based on potentiometric data, as clearly visible from Figure 6B. The application of PAR in an analogous experiment indicated that this probe virtually outcompetes only PC3 for Zn(II) (Figure 7B). PC2 is too weak, while PC4 is too strong a ligand for the competition, and the formation constant of PC3 was only calculated following the procedure presented by Kocyła et al.^64^ The determined log *K*^7.4^ value for the ZnPC3 complex is convergent with the CI^7.4^ value, indicating that spectroscopic competition and potentiometry are useful methods for the affinity determination of such ligands.

### Isothermal Titration Calorimetry

**Figure 8:**
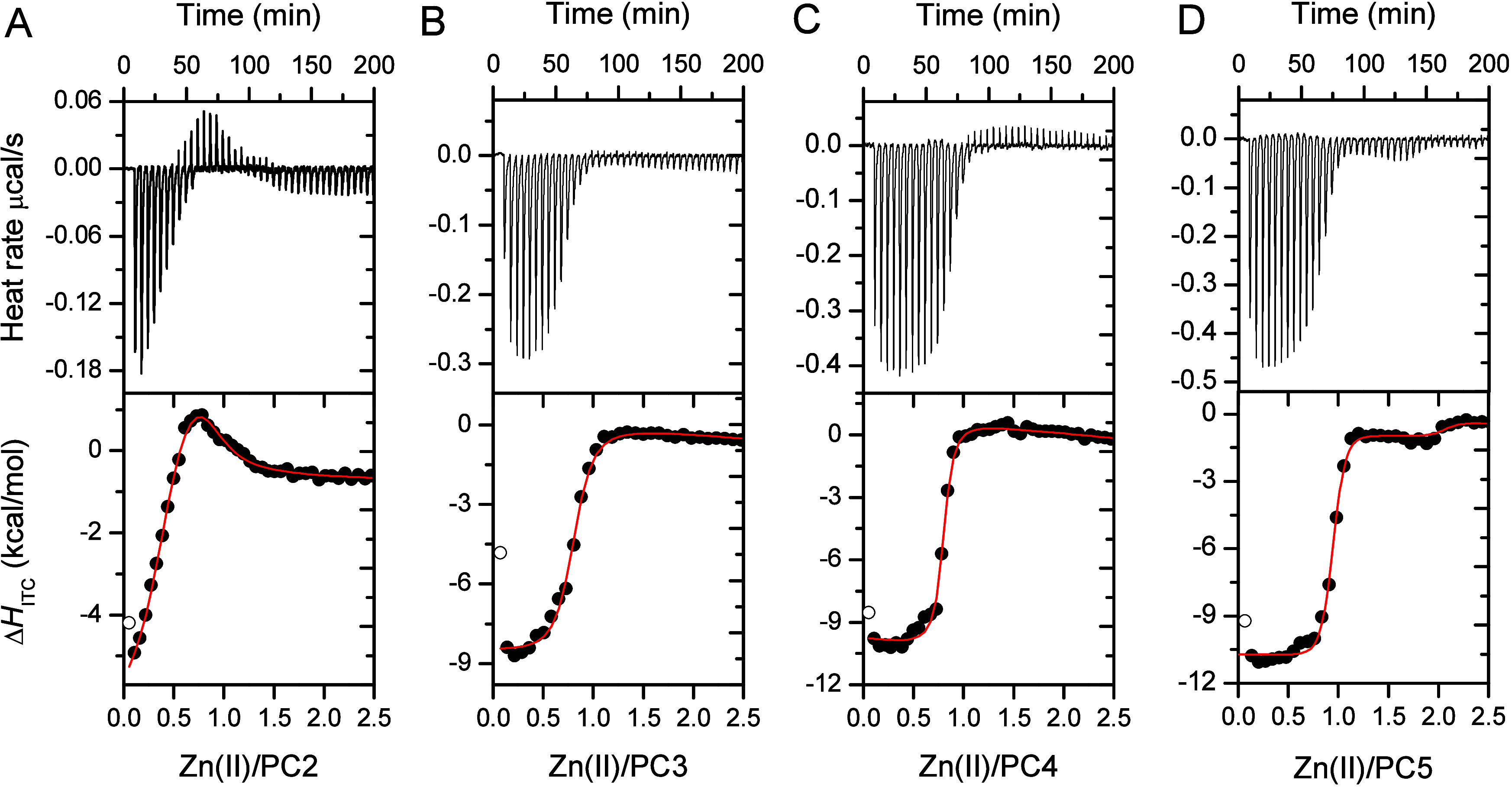
ITC titrations/profiles of Zn(II)-titrated PC2 (A), PC3 (B), PC4 (C), and PC5 (D) in 50 mM HEPES, pH 7.4, 100 mM NaCl, 600 μM TCEP. Concentration of peptides (titrate) was 50 μM while ZnSO_4_ (titrant) was 0.5 mM. Top panel represents heat rates for particular PC titration, while bottom panel represents Δ*H*_ITC_ in kcal/mol fitted to competitive (PC2) and independent model (PC3–PC4).

The investigation of the thermodynamic properties of d-block metal complexation by proteinaceous systems, which form highly stable complexes, presents several limitations that often result in underestimated formation constants.^3,49,66,67^ Therefore, our objective was limited to evaluating the stoichiometries of complex species formed in solution and assessing the enthalpies derived from isothermal titration calorimetry (Δ*H*_ITC_). To minimize the impact of air sensitivity in the investigated Cys-rich systems, all experiments were conducted in excess of tris(2-carboxyethyl)phosphine (TCEP).^48^ Titration profiles indicate that only PC2 forms a biscomplex, which dissociates into equimolar species upon the addition of additional Zn(II) ions (Figure 8A). This complex rearrangement is energetically unfavorable (the Δ*H*_ITC_ of this process is positive) when considering only the enthalpic factor. PC3–PC5 peptides form equimolar complexes, with Δ*H*_ITC_ values significantly lower than those of PC2 (Figure 8B–D; Table 5). However, for PC4 and PC5, Δ*H*_ITC_ remains almost identical, confirming observations from the stability studies. Furthermore, the profile of PC5 titration indicates an additional binding site for two Zn(II) ions per PC5 molecule, which can be observed by a very weak Δ*H*_ITC_ change.

**Table 5:** Thermodynamic Parameters Obtained from ITC Experiments for Zn(II)-PC Systems^a^

peptide	complex formation	Δ*H*_ITC_ (kcal/mol)	*n*_H_	Δ*H*° (kcal/mol)	Δ*G*°	–*T*Δ*S*° (kcal/mol)
PC2	Zn(PC2)_2_	–6.47 ± 0.21	3.88	12.99	–9.09	–22.09
	ZnPC2	4.39 ± 0.42	2.94	19.14	–9.09	–28.24
PC3	ZnPC3^b^	–8.97 ± 0.47	2.89^b^	5.52	–11.47	–17.00
	ZnPC3^c^	–8.97 ± 0.47	3.89^c^	9.96	–11.47	–15.23
PC4	ZnPC4	–10.31 ± 0.76	3.90	9.30	–14.93	–24.22
PC5	ZnPC5	–10.62 ± 0.65	3.90	8.96	–15.58	–24.54

a All titrations were performed at 25 °C in 50 mM HEPES buffer, 0.1 M NaCl, and 600 μM TCEP. *n*_H_ denotes number of protons dissociated from ligand(s) upon binding of one Zn(II) and was calculated based on potentiometric data using HySS software.^53^ Errors are presented only for fitted values (here Δ*H*_ITC_).

b Considering species with protonated amine group and {3SO} coordination sphere.

c Considering species with deprotonated amine groups and {3SN} coordination sphere.

To dissect ITC enthalpies and obtain more information regarding Zn(II) complexation, the protonation status of the metal anchoring residues of PCs was analyzed as a first step. Zn(II) complexation by Cys-containing peptides is accompanied by thiol (or amine group) deprotonation; therefore, the experimental enthalpy (Δ*H*_ITC_) must be corrected for the heat of protonation of the buffer, as indicated in eq 2.^66,67^ Considering the p*K*_a_ values of PCs and their assignment to particular groups discussed in our and other studies, the number of protonated residues at pH 7.4 was calculated using HySS software (Table 5).^34,37,38^ Note that, in the case of the Zn(PC2)_2_ species, two peptide molecules with four Cys residues participate in Zn(II) binding and were considered during the *n*_H_ calculation. In the case of ZnPC2, only two Cys residues and an additional amine group {2SNO} were considered during *n*_H_ calculation. In the case of ZnPC3, only Cys residues were considered {3SO} during the *n*_H_ calculation, or additionally, deprotonation of the amine group {3SN} was involved in this process (Table 5).
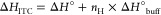
2Δ*H*° represents the buffer-independent intrinsic reaction enthalpy, *n*_H_ denotes the number of protons dissociated during Zn(II) complexation, and Δ*H*°_buff_ signifies the buffer-specific heat of protonation, with a value of −5.02 kcal/mol for HEPES.^68^ Additionally, the entropic component of the complexation reaction (−*T*Δ*S*°) was calculated based on Gibbs’ law (eq 3).
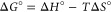
3

This equation utilizes Δ*G*° values calculated based on the competivity indexes of Zn(II)-PC complexes from Table 4, as indicated in eq 4. Given that the formed complexes are tight or very tight, the formation constants determined from ITC titrations would be underestimated (as discussed earlier). In the thermodynamic analysis of such systems, it is more appropriate to utilize actual stability data determined by a reliable method (such as potentiometry or competition studies). These calculated Δ*G*° values describe the complexation energy at a constant pH and are free from energetic effects related to the buffer component protonation.
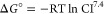
4

All calculated parameters are presented in Table 5 and demonstrate that the Zn(II) coordination by PCs is significantly driven by entropy changes.

## Discussion

### Zn(II) Complexation Properties of PC2-PC5

As demonstrated in previous studies on Cd(II) complexation, phytochelatins are highly dynamic peptides capable of forming various bis-, mono-, and polynuclear complexes with weakly structured polypeptide chains. The Zn(II) complexes investigated in this study are similarly highly dynamic, although only bis- and monomeric species predominate, depending on the peptide. No polynuclear species are formed, which is one of the major differences, besides stability, between both metal ions. Only in the case of PC5 was a minor presence of binuclear species observed. The major Zn(II) donor in all complexes is sulfur, which is consistent with its preferences. In the excess of PC2, two peptide molecules are involved in Zn(PC2)_2_ complex formation at neutral pH (Figure 2, Figure S6). Co(II) data indicate the tetrahedral geometry of the complex, and it is therefore likely that this will also be the case for Zn(II). ZnH_2_L_2_, ZnHL_2_, and ZnL_2_ complexes are formed at higher pH, all with tetrathiolate coordination (Table 2, Figure 3, Figure 5). At a very basic pH, a ZnH_–1_L_2_ species is formed, where a deprotonated water molecule (OH^–^) is involved in Zn(II) binding. The tetrathiolate coordination sphere is present only in the biscomplex of PC2, while in the case of ZnPC2 (Figure 3), the coordination sphere remains unsolved; however, based on previous NMR studies with Cd(II), we may anticipate the formation of ZnHL species where the amine group remains protonated with more likely a {2S2O} sphere and ZnL, where the amine group is deprotonated and involved in Zn(II) binding {2SNO}.^34^ Since PC3 does not form a biscomplex, tetrathiolate coordination is not possible to achieve, as nicely probed by Co(II) titration (Figure 2, Figure S6). Similarly to the monomeric ZnPC2 complex, in this case, two types of complexes are more likely formed at a neutral pH: ZnHL with {3SO} and ZnL with {3SN} (Figure 3). At more acidic conditions, more protonated species were found with more likely lower sulfur donors (Table 2, Figure 5). A fully tetrathiolate sphere in an equimolar complex is achieved starting from PC4 (Figure 3). The high flexibility of the peptide chain, mainly due to γ-peptide bonds, allows enough structural freedom for four thiol groups’ complexation at once at neutral pH. However, it should be mentioned that more protonated Zn(II) complexes are also formed at lower pH, where Zn(II) is bound by a lower number of sulfur donors (Table 2, Figure 5). PC5 remained very similar to PC4 in Zn(II) complexation in all performed studies, both structural and thermodynamic. It forms a tetrathiolate complex (ZnHL and ZnL) at neutral pH, while at lower pH, variously protonated species are also present (Table 2, Figure 3, Figure 5). The ITC profile for PC5 indicated the formation of Zn_2_PC5 stoichiometry, where an additional metal ion is bound to the fifth Cys residue and available donors. A very low Δ*H*_ITC_ suggests that only a small fraction of that complex is formed under the used conditions. The blue shift present in UV–vis spectra at higher Zn(II) concentrations confirms this PC’s tendency to form dimeric species (Figure 1). Although greater than PC5 peptides were not investigated here, based on Cd(II) studies, we may expect a higher stability of the Zn_2_PC6 complex than for Zn_2_PC5. Whether this complex has a chance to be formed in live organisms is questionable since additional Zn(II) would be bound to lower PCs (discussion below).

### Thermodynamics of Zn(II)-PC System

Although Cd(II) complexes of PCs have been extensively studied, little is known about Zn(II) interactions and even less about the thermodynamics of Zn(II)-PC systems. Only one paper is devoted to the stability determination of the Zn(II)-PC4 complex using ITC.^43^ The reported formation constant of 8.8 × 10^5^ M^–1^ (log *K* = 5.9) is 5 orders of magnitude lower than that determined here in potentiometric studies (Table 4). As discussed earlier, the ITC of tight metal-peptide/protein interactions frequently underestimates the formation constant. Therefore, our main goal was to perform systematic studies of the PC2–PC5 series’ interaction with Zn(II) using the same method that allows for the determination of stability constants in a wide range of affinities, namely, potentiometry. The obtained values were later compared with those from competitive studies with Zincon and PAR, confirming the reliability of the potentiometry. Another goal of our studies was to determine how the stabilities of Zn(II)-PC complexes change with the number of γ-GluCys repeats in the peptide; therefore, in some studies, GSH with one repeat was used. Finally, we aimed to answer the question of what factors drive the stability of Zn(II)-PC complexes and how they are similar to or different from Cd(II) ones.

The initial observation that Zn(II) complexes with PC2 are considerably weaker than those with other PCs was made during the spectroscopic titrations. The absence of peptide saturation suggests that the affinity of PC2 toward Zn(II) falls within the micromolar range (Figure 1). Furthermore, pH-dependent spectroscopic titrations of the GSH-PC5 system with Zn(II) revealed a significant shift in the isotherms by 1.7 orders of magnitude (Figure 4A,B). Such a substantial shift has been shown to correlate with several orders of magnitude difference in the formation constants of Zn(II) complexes with Cys-containing peptides.^3,69^ The comparison of the competivity index with p*K*_a_′ values indicates nonlinear relationships (Figure 4C), contrasting with the previously investigated Cd(II)-PC system. This nonlinearity is more likely due to the formation of heteroleptic Zn(II) complexes where donors other than thiolates, {O, N}, may participate in Zn(II) binding (GSH, PC2, and PC3), in contrast to the Cd(II) system, where tetrathiolate species are predominant.^37^ Nonetheless, the significant shift of p*K*_a_′ values suggests that the stability of Zn(II) complexes increases from GSH to PC4. The stability of the Zn(II) species with PC5 is almost identical with that of PC4.

The potentiometric data obtained in this report (Table 1, Table 2) indicate the formation of multiple complexes, making a direct comparison challenging without considering ligand protonation. In this scenario, only rough comparisons of constants for the same types of complexes, such as all ZnHL and all ZnL, are possible. Figure 6A illustrates an increase of approximately 12 and 11 orders of magnitude for ZnHL and ZnL complexes (Table 2), respectively, showing an almost linear increase in the stability of these particular complexes from GSH to PC4, with comparable constants for PC4 and PC5. The evaluation of speciation profiles and the assignment of stability constants over a wide pH range allowed us to translate them into competivity indexes for direct comparison between affinities assigned to different peptides. The calculated CI^7.4^ values demonstrate micro- and submicromolar affinities for GSH and PC2, respectively, confirming observations from spectroscopic studies. The affinity of the Zn(II)-PC3 system is in the nanomolar range, while it is in the picomolar range for the Zn(II)-PC4 and Zn(II)-PC5 systems. The stability difference between the weakest (GSH) and the tightest complex (PC4, PC5) is approximately 6 orders of magnitude, which corresponds to a ΔΔ*G*° of around −9 kcal/mol (Table 5). This suggests that elongating the peptide length by one γ-Glu-Cys segment results in an increase in stabilization Gibbs free energy (Δ*G*° value decreases) by approximately −3 kcal/mol. The increase in stability is not caused by the metal-coupled folding process, which contributes significantly to the complexation enthalpy in the structural folds. All PCs do not form stable secondary or tertiary structures upon Zn(II) coordination, unlike some zinc finger motifs with femtomolar affinity.^3,60^ Therefore, metalation processes resulting in Zn(II) binding to the tetrathiolate environment in the nano- or picomolar range are further modulated by multiple effects, as seen in the case of metallothioneins.^7,70−72^ Seven Zn(II) ions bind to mammalian MT2 within this range of affinities, modulated by electrostatic effects and domain proximity.^73^ Regardless of these effects, the maximal affinity (minimal dissociation constant) of tetrathiolate sites without metal-coupled effects is in the low picomolar range. Zn(II) affinity for PC4 and PC5 falls exactly within this range. The weaker affinity of PC3, PC2, and GSH for Zn(II) can be attributed to a different composition of the coordination sphere or stoichiometry related to the enthalpic and entropic effects of Zn(II) complexation processes. The molecular reasons for the increased stability in the phytochelatin series are granted only in part by enthalpy-related factors due to the increasing number of sulfur donors in the peptide resulting in decreasing Δ*H*° in the series. The complexation of PCs is mainly driven by entropic-related factors. Two contrasting phenomena significantly influence the entropy dynamics within PCs: the chelate effect and conformational restriction. The chelate effect contributes to entropy through stoichiometric and structural adjustments. Longer PCs tend to form more constrained Zn(II) complexes, leading to an unfavorable entropy change. Nevertheless, the dominant influence appears to be the chelate effect, outweighing the relatively smaller energies associated with the conformational restriction. Moreover, the notable increase in affinity observed in PC4 and PC5 may stem from their inherent ability to initially form tetrathiolate M(II) species, unlike that of shorter PCs.

### In Search of a Biological Role of Zn(II)-PC Species

The results presented here demonstrate that PCs bind Zn(II) more efficiently than previously thought. Although the shortest PC2 binds Zn(II) with submicromolar affinity, the longer ones bind Zn(II) in the nano- to picomolar range. This contrasts with previous data showing that PC2 and PC3 bind Zn(II) with micromolar affinity, differing from ours by up to 6 orders of magnitude.^42,43^ The actual range of Zn(II)-PC species covers the concentration range of exchangeable Zn(II) found in eukaryotic cells, raising the question of whether PCs can bind Zn(II) in living organisms and what the biological role of such complexes is. It is worth noting that the broad picture becomes even more complicated when the presence of abundant oxygen- and nitrogen-based chelator LMWLs is taken into account. However, their Zn(II) binding affinities are on average a few orders of magnitude lower than those assigned to GSH, which serves as a precursor of PC synthesis. Therefore, their Zn(II) complexes can be easily outcompeted by any of PC homologues.^13,14^ Our results are supporting a role of PCs both in buffering and in muffling cytosolic Zn(II).^6^ Using more sensitive analyses, it has been demonstrated that relatively short PC peptides (mainly PC2–PC4) are present in plant cells not exposed to an excess of toxic metal ions such as Cd(II).^17^ On the other hand, it is known from studies on a number of different plant species that Zn(II) exposure may activate the synthesis of PCs of various lengths, albeit generally to a lower extent than upon Cd(II) exposure.^74^ However, the efficiency of PC synthesis activation is difficult to compare, since externally applied concentrations do not necessarily correlate with actual exchangeable Zn(II) concentrations in the cytosol. When metal concentrations causing the same degree of growth inhibition were applied to the *Arabidopsis thaliana* plant, Zn(II) treatment resulted in PC levels around 30% of those found after Cd(II) exposure.^75^ This indicates that Zn(II) efficiently activates PC synthesis and explains the Zn(II) tolerance effects of PCs, i.e., the muffling activity, that can be inferred from the Zn(II) hypersensitivity of mutants defective in PC synthesis^24,74^ as well as the Zn(II) tolerance gains resulting from PCS overexpression.^76^

**Figure 9:**
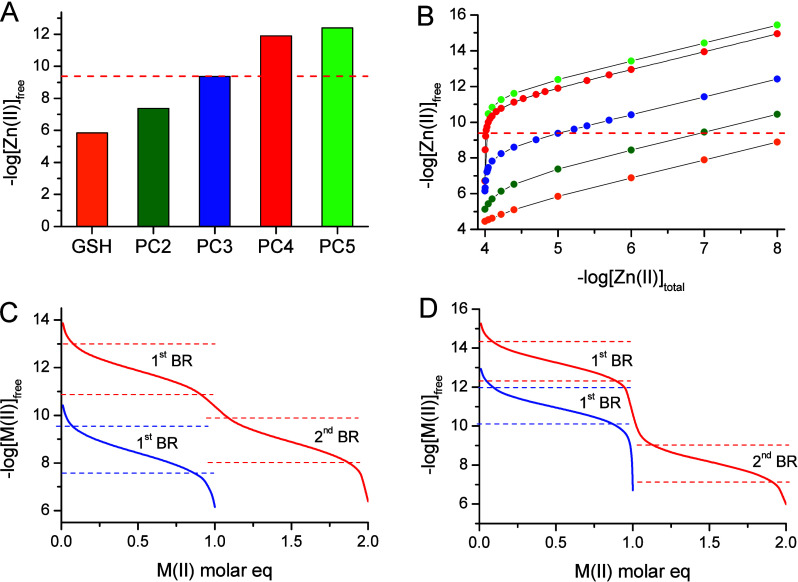
Speciation and free Zn(II) analysis in GSH-PC5 series at pH 7.4 based on stability constants obtained in this and previous studies.^37,62^ (A) Free Zn(II) concentrations as a result of the complexation of 0.1 mM Zn(II) with the 1 mM ligand. Red dashed line indicates exchangeable cellular Zn(II) concentration found in *Arabitopsis thalina* root cells.^15^ (B) Relations between total and free Zn(II) concentrations of systems containing 0.1 mM ligand and increased concentration up to 0.1 mM of Zn(II). Colors and dashed red line correspond to those from A. (C and D) Comparison of free Zn(II) (blue line) and free Cd(II) (red line) as a result of 0.1 mM PC3 (C) and PC4 (D) complexation from 0 to 1 (Zn(II) case) or 0–2 (Cd(II) case) molar equiv. Red and blue dashed lines indicate the free metal buffering ranges (BRs). M stands for Zn(II) or Cd(II).

Free Zn(II) in simulations plotted in Figure 9 does not precisely correspond to the exchangeable Zn(II) concentration. In cells, all Zn(II) ions of the exchangeable zinc pool are complexed by LMWLs (they are not free), including PCs. However, free Zn(II) in simulations informs that PCs contribute to the cellular zinc pool by comparing its value with the exchangeable Zn(II) concentration in root cells. Figure 9A shows the simulation of free Zn(II) concentrations maintained by the series of GSH-PC5 based on determined stability constants. The profile of decreased free Zn(II) concentrations buffered from the micro- to low picomolar range by these peptides has a tendency highly similar to that observed for the comparison of stability data (Figure 6). Calculations performed in Figure 9A, only for a one molar ratio, indicate that PCs could be involved in the buffering of Zn(II) concentration since the determined exchangeable Zn(II) concentration in *Arabidopsis* root was found to be 400 pM, as indicated by the red dashed line.^15^ To determine how free Zn(II) concentrations vary at various Zn(II)/peptide molar ratios, we conducted appropriate calculations for the GSH-PC5 series. Figure 9B illustrates how free Zn(II) concentrations behave as a function of the total Zn(II) present in the system, ranging from 10^–8^ to 10^–4^ M. It is evident that, depending on molar ratios, various PCs may participate differently in Zn(II) binding. This Zn(II) buffering may occur within the range of hundreds of pM of exchangeable Zn(II) concentration, but it may also extend to higher and lower ranges. PC4 and PC5 buffer Zn(II) within the hundreds of pM range at ratios near equimolar, while PC2 and PC3 do so at higher peptide-to-Zn(II) molar ratios. It is intriguing that GSH appears to be a poor ligand for Zn(II) buffering; however, its high concentration in cells still warrant the formation of Zn(II)-GSH complexes required for certain processes, such as Zn(II) transfer, even at low complex concentrations.

Although Zn(II)-PCs and Cd(II)-PC systems demonstrate substantial similarities in trends of affinity increase with the number of γ-Glu-Cys repeats, they differ to a certain degree in the stoichiometry of the formed complexes. Both Zn(II) and Cd(II) form bis-complexes for PC2 and equimolar complexes for PC2–PC5; however, only Cd(II) complexes have a tendency to form polynuclear species. This difference arises from the natural tendency of Cd(II) to form tetrathiolate coordination spheres. Therefore, when its concentration exceeds PC, binuclear complexes start to form, where cysteine sulfur donors become bridging between two Cd(II) ions. Such a way of Cd_*x*_(Cys)_*y*_ species formation is well-known for metallothioneins of different organisms, where domain architecture is organized in such a way that each Cd(II) is bound by four sulfur donors (occasionally nitrogen donors from His residues) in clusters of different sizes.^77−79^ Although Zn(II) also forms clusters within metallothioneins, it does not tend to do so with phytochelatins. A slight tendency for binuclear complex formation was observed by ITC only for the longest peptide. Neither PC2, PC3, nor PC4 form binuclear species with Zn(II), while all of them form complexes with Cd(II). This chemical feature distinguishes PCs by their metal binding and capacity. This observation would not be possible without previous and current careful investigation of both systems with a set of the same or similar techniques. Figure 9C compares the Zn(II) saturation of PC3 up to 1 molar equiv with Cd(II) saturation up to 2 molar equiv with respect to exchangeable metal concentrations. As pointed out above, PC3 is able to buffer Zn(II) from −log[M(II)]_free_ ∼8 to ∼10, while Cd(II) binding is a two-step process. In the first step, only equimolar complexes are formed, and buffering occurs from ∼11 to ∼13. In the second step, polynuclear species are formed, and buffering occurs from ∼8 to ∼10. Figure 9D demonstrates a similar comparison for PC4, where buffering ranges are adequately elevated due to stability differences. Since PCs can bind two times more Cd(II) compared to Zn(II), even for short PCs, there is a possibility for the lack of reduced production of longer PCs with the same Cd(II) binding capacity. In fact, long PCs are rarely observable in plants and only under prolonged or heavy Cd(II) exposure, because shorter PCs serve as substrates for the synthesis of longer PCs. Many reports show that PC2–PC5 plays the most important role in Cd(II) detoxification and storage. Therefore, we postulate that, in the case of Cd(II), PCs behave differently from Zn(II). They bind metal ions, detoxifying cells from Cd(II), and if its concentration increases, they can store its excess without the necessity for longer PC production. Our studies indicated that longer chains have a similar affinity for metal ions, so continuous production will not increase their affinity, only binding capacity, but it requires time and energy.

Zinc is an essential metal ion that has an impact on many cellular processes. Its exchangeable cellular concentrations are controlled by transporters, vacuole systems, and metallothioneins (MTs). Under normal conditions, not exposed to Zn(II) excess, PCs may participate in zinc buffering, offering Zn(II) for proteins that need it or accepting excess similar to MT systems. Participating PCs in such processes would rely on the documented basal level of PCs synthesized under normal conditions, that is, in the absence of any excess metal excess. Such data are hardly available since most investigations correspond to stressed conditions or are not clearly shown in published articles. However, there are few reports that may value that issue. Data published by Ahner et al. consistently showed the presence of PCs in phytoplankton sampled from the Atlantic, indicating ubiquitous synthesis of PCs in low-metal environments.^80^ In another one, PC2 levels determined in *Arabidopsis thaliana* roots and *Chlamydomonas reinhardtii* under control conditions are usually in the range of 5 to 30 nmol/g fresh weight.^81,82^ These amounts correspond, on average, to the micromolar range of PC2. Furthermore, PCs’ buffering role would be consistent with the lower Zn(II) levels observed in two independent experimental settings for the leaves of plants deficient in PC synthesis.^24^ In both cases, *Arabidopsis thaliana* PCS1 mutants showed about 30% lower zinc accumulation. Given the tight control over zinc homeostasis, this reduction represents a remarkable effect. Few other proteins have been demonstrated to influence leaf zinc accumulation to a similar extent. It has been hypothesized that this leaf zinc phenotype is due to a reduced availability of Zn(II) for root-to-shoot translocation, which at least in part is dependent on the cytosolic Zn(II) buffering capacity of root cells.^24^

## Conclusions

This study demonstrates that short phytochelatins (PC2–PC5) form highly dynamic complexes with Zn(II) that lack a secondary structure. Zn(II) forms bis- and equimolar complexes with the investigated PCs without the formation of binuclear species, which contrasts with Cd(II) complexes. Depending on the species, Zn(II) is bound either in a tetrathiolate environment or in a mixed coordination sphere involving oxygen and nitrogen donors. Potentiometric and competition studies indicate that the affinity for Zn(II) increases linearly from PC2 to PC4, ranging from the micro- to low-picomolar range. Elongating the peptide chain to PC5 does not significantly increase the stability of the complex formed complex. Calorimetric investigations demonstrate that the observed stability elevation is primarily driven entropically due to the chelate effect and conformational restriction and to a lesser extent enthalpically due to the increasing number of sulfur donors. Our results indicate that the observed affinity of the investigated PCs falls within the range of exchangeable Zn(II) concentrations (hundreds of pM) observed in plants, supporting the role of PCs both in buffering and in muffling cytosolic Zn(II) concentrations under normal conditions, not exposed to zinc excess, where short PCs have been identified in numerous studies. Furthermore, we found that Cd(II)-PC complexes demonstrate significantly higher metal capacities due to the formation of binuclear species, which are lacking in the Zn(II)-PC system. This supports the role of PCs in Cd(II) storage and Zn(II) buffering and muffling. Received data are of high importance for zinc biology and understanding molecular bases of detoxification mechanisms of Cd(II) opening a door for future studies.

## Supporting Information Available

ESI-MS spectra of Zn(II) complexes of the series of PCs acquired in 10 mM (NH_4_)_2_CO_3_ (pH ∼ 8) at different Zn(II):PC ratios; raw spectra of spectroscopic titrations of 10 μM PCs with 0.5 mM ZnSO_4_ at pH 7.4; visible range of spectroscopic titrations of 250 μM PCs with 50 mM Co(NO_3_)_2_ at pH 7.4; Zn(II)-PC2 species distribution plotted for 250 μM Zn(II) and 500 μM of PC2; Zn(II)-GSH species distribution plotted for 500 μM Zn(II) and 500 μM of GSH and 250 μM Zn(II) and 500 μM of GSH; spectroscopic competition between 100 μM Zincon and reduced glutathione (GSH) (PDF)
