# Supplementary material for: Phytochelatins Bind Zn(II) with Micro- to Picomolar Affinities without the Formation of Binuclear Complexes, Exhibiting Zinc Buffering and Muffling Rather than Storing Functions
Source: Inorg Chem. 2024 Jun 7;63(24):10915–31. doi: 10.1021/acs.inorgchem.4c01707 (PMC11191002; doi:10.1021/acs.inorgchem.4c01707)
Supplement: Supplementary file 1 — ic4c01707_si_001.pdf [file ic4c01707_si_001.pdf]

Supplementary information

Phytochelatins bind Zn(II) with micro- to picomolar affinities without the formation of binuclear complexes, exhibiting zinc buffering and muffling rather than storing functions

Marek Łuczkowski,[1] Weronika Leszczyńska,[1] Joanna Wątły,[1] Stephan Clemens[2] and Artur Krężel[1]*

[1]*Department of Chemical Biology, Faculty of Biotechnology, University of Wrocław, Joliot-Curie 14a, 50-383 Wrocław, Poland*

[2]*Department of Plant Physiology, Faculty of Biology, Chemistry and Earth Sciences, University of Bayreuth, 95440 Bayreuth, Germany*

Present Addresses:

†Faculty of Chemistry, University of Wrocław, Joliot-Curie 14, 50-383 Wrocław, Poland

Corresponding Author:

*Artur Krężel, e-mail: artur.krezel@uwr.edu.pl

Keywords: Cadmium toxicity; Free zinc; Exchangeable zinc; Isothermal titration calorimetry (ITC); 4-(2-pyridylazo)resorcinol (PAR); Labile zinc; Plant zinc; Zinc homeostasis.

Table S1. Experimental and theoretical monoisotopic mass values of synthesized peptides and their sequences.

Peptide	Peptide sequence	MW_{cal}	MW_{exp}
PC2	γEC-γEC-G	539.1	539.3
PC3	γEC- γEC-γEC-G	771.2	771.3
PC4	γEC- γEC-γEC- γEC-G	1003.2	1003.3
PC5	γEC- γEC-γEC- γEC-γEC-G	1235.3	1235.6

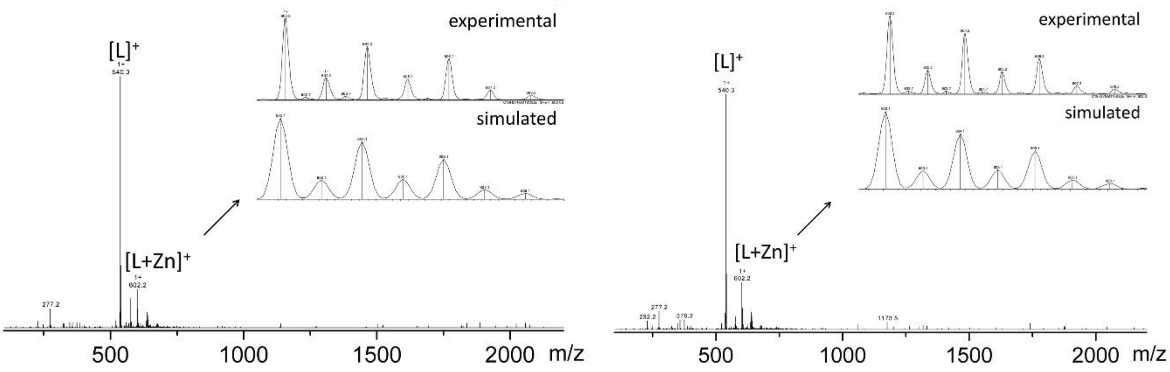

Figure S1. Mass spectra of Zn(II):PC2 system in 10 mM $(NH_4)_2CO_3$ (pH ~8) at ratios 50 μM:100 μM (left panel) and 50 μM:50 μM (right panel), respectively.

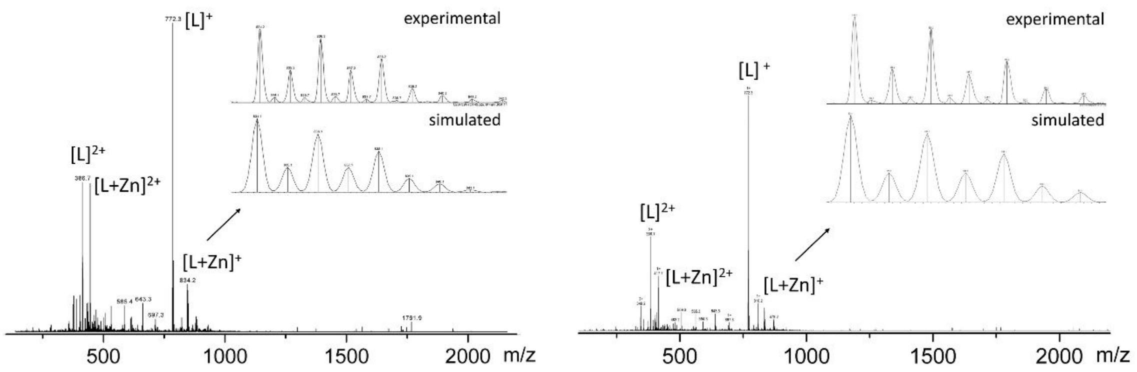

Figure S2. Mass spectra of Zn(II):PC3 system in 10 mM $(NH_4)_2CO_3$ (pH ~8) at ratios 50 µM:100 µM (left panel) and 50 µM:50 µM (right panel), respectively.

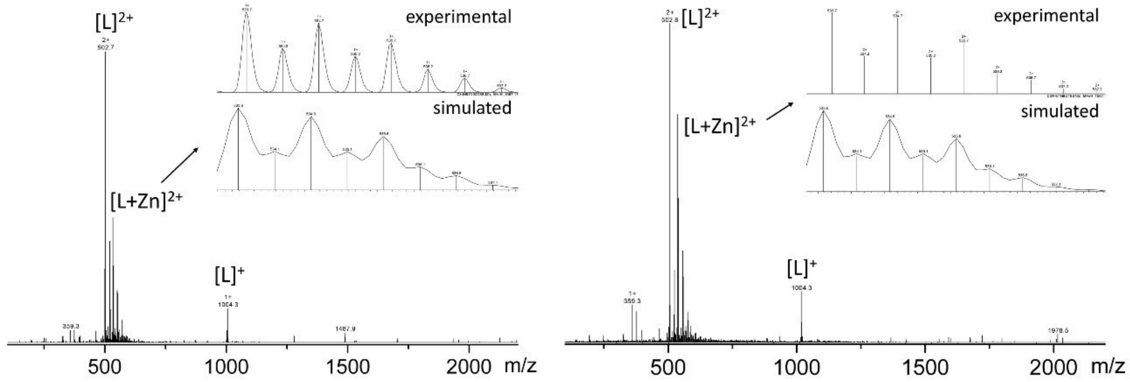

Figure S3. Mass spectra of Zn(II):PC4 system in 10 mM $(NH_4)_2CO_3$ (pH ~8) at ratios 50 µM:100 µM (left panel) and 50 µM:50 µM (right panel), respectively.

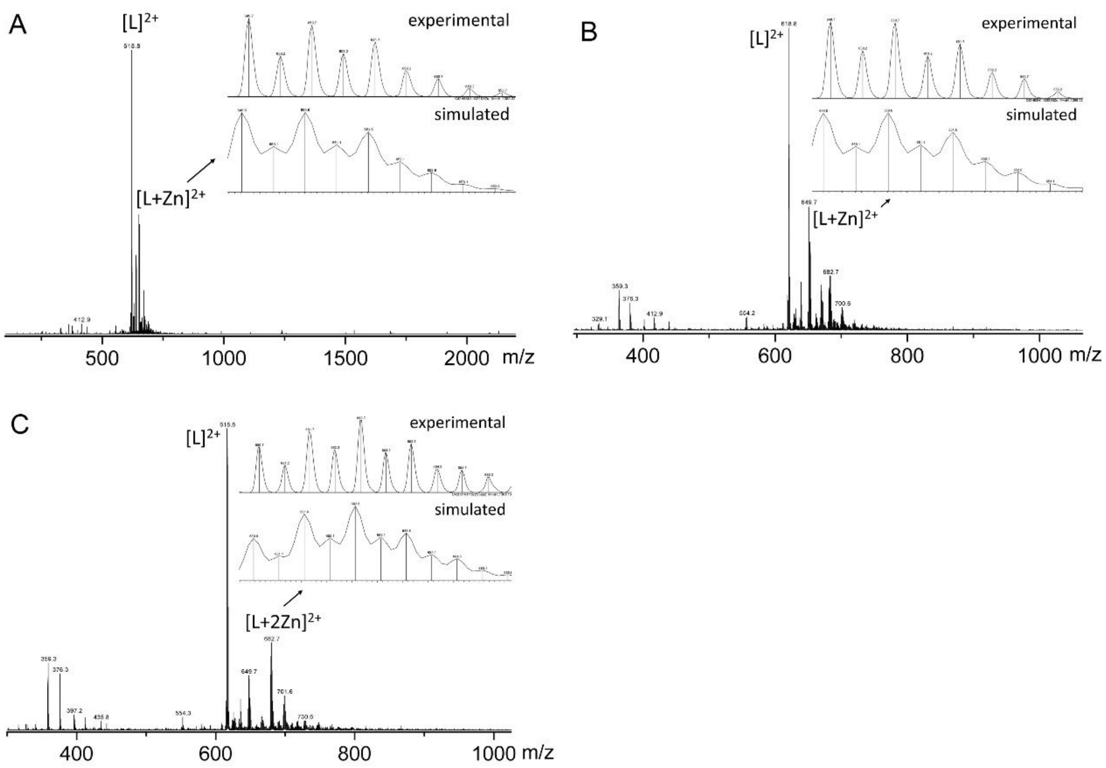

Figure S4. Mass spectra of Zn(II):PC5 system in 10 mM $(NH_4)_2CO_3$ (pH ~8) at ratios 50 µM:100 µM (top left panel), 50 µM:50 µM (top right panel), and 100 µM: 50 µM (bottom panel), respectively.

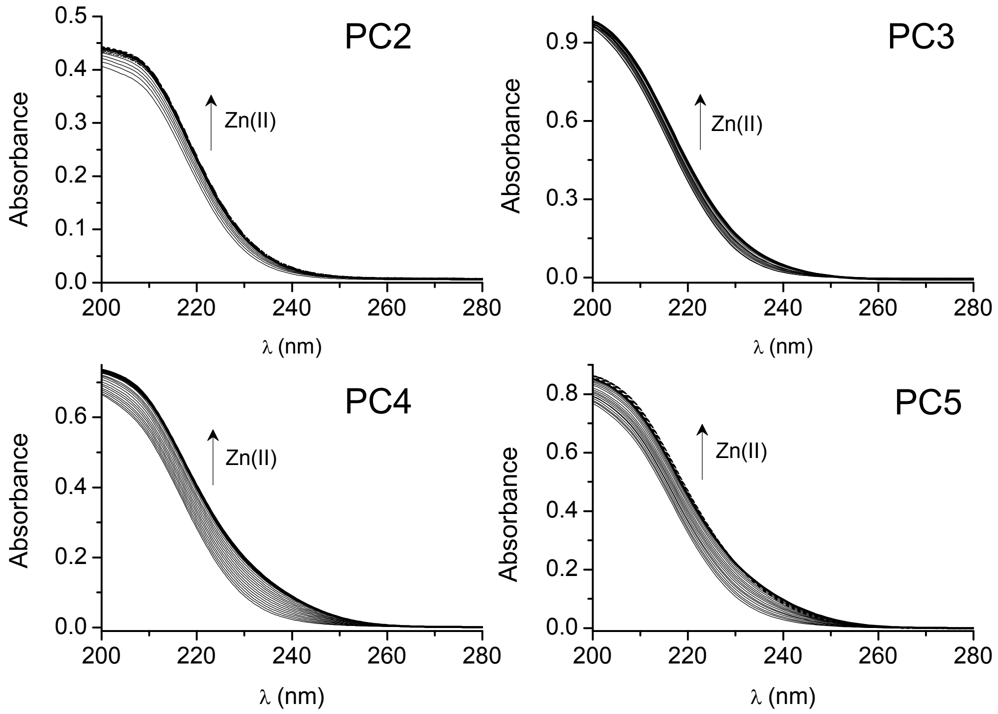

Figure S5. Spectroscopic titrations of 10 μM PC2-PC5 series with $ZnSO_4$ in 10 mM Tris-HCl buffer pH 7.4 (I = 0.1 M from $NaClO_4$) with 0.08 mM TCEP.

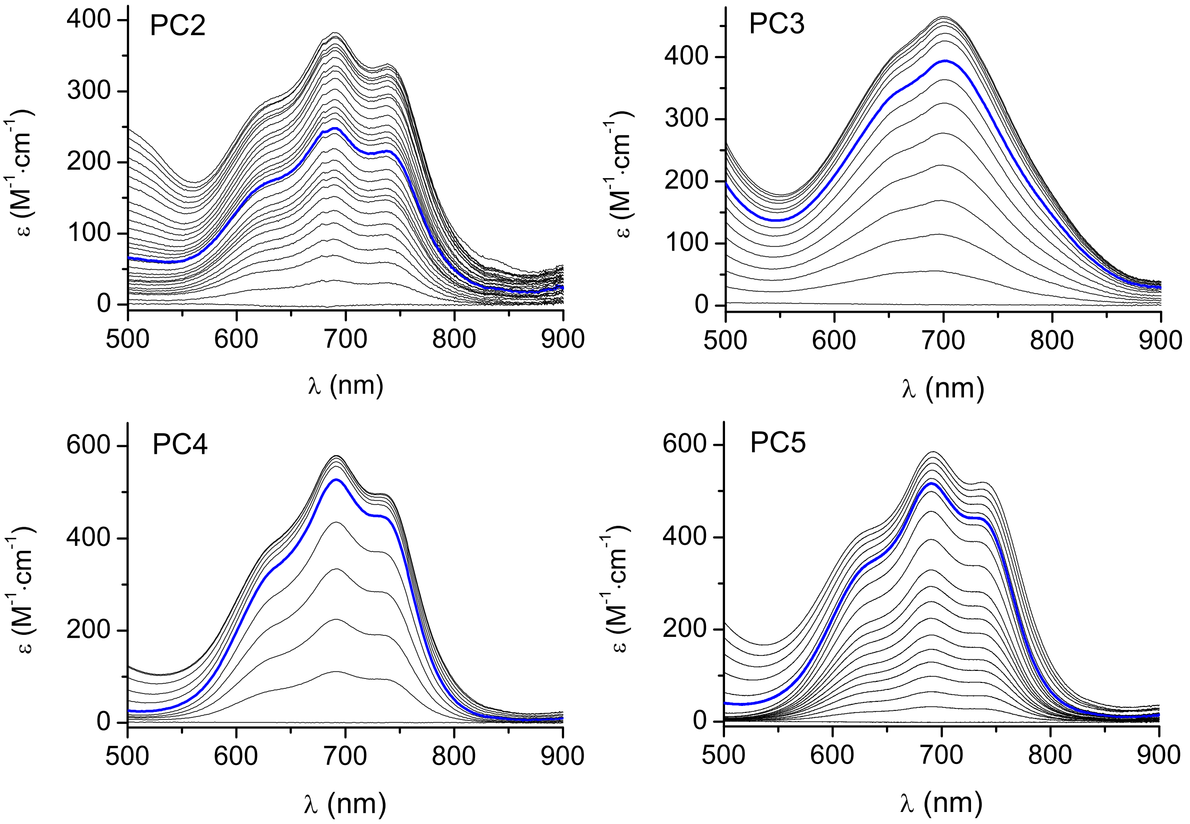

Figure S6. Spectroscopic titrations of 250 μM PC2-PC5 with 50 mM $Co(NO_3)_2$ in 20 mM TES buffer pH 7.4 (I = 0.1 M from NaF). Blue spectra are for 1 molar ratio of Co(II)-to-PC.

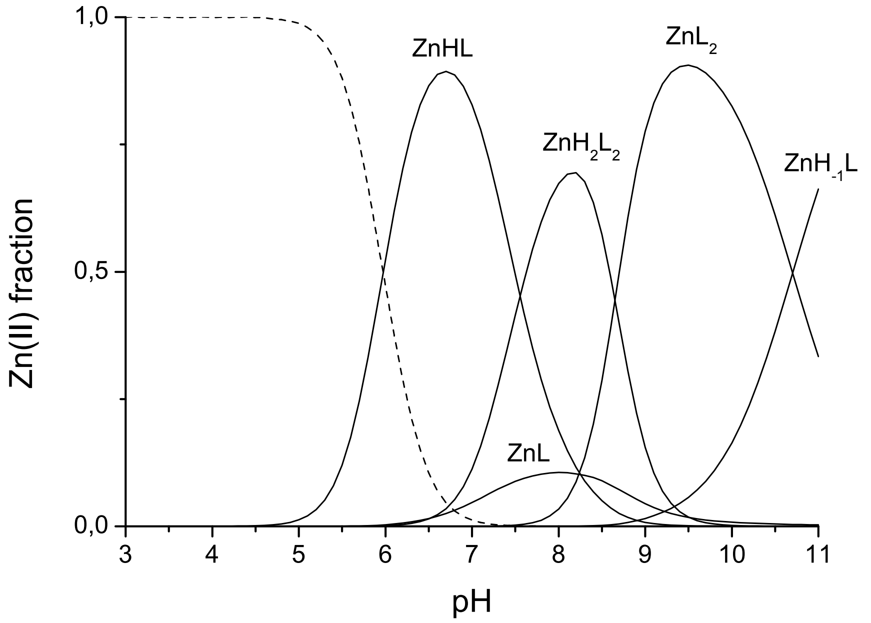

Figure S7. Zn(II)-PC2 species distribution plotted for 250 μM Zn(II) and 500 μM of PC2.

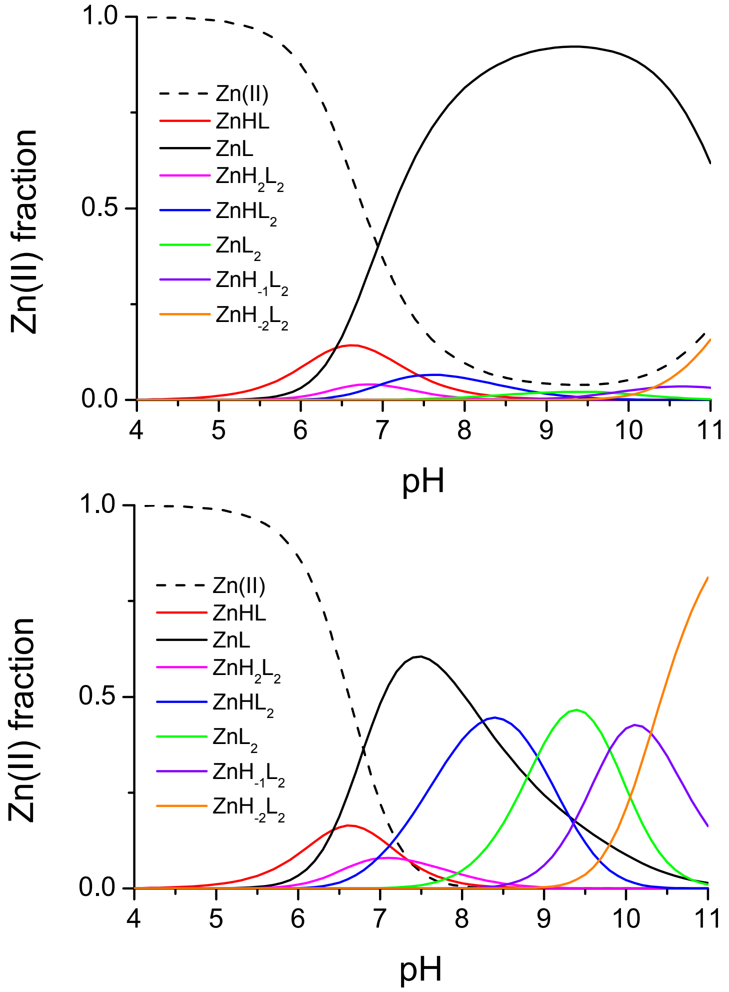

Figure S8. Zn(II)-GSH species distribution plotted for 500 μM Zn(II) and 500 μM of GSH (top) and 250 μM Zn(II) and 500 μM of GSH (bottom).

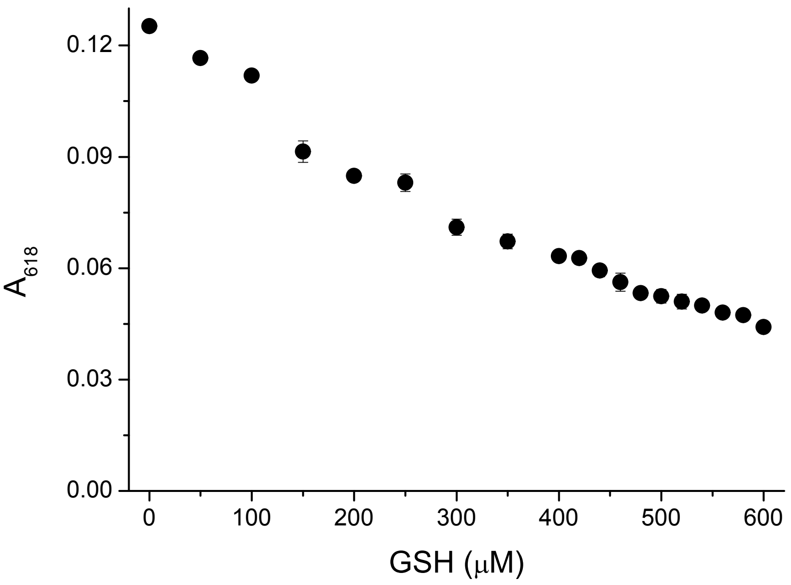

Figure S9. Spectroscopic competition between 100 μM Zincon and reduced glutathione (GSH). Zincon was initially saturated with 5 μM $ZnSO_4$ in 50 mM HEPES buffer, 100 mM NaCl, 100 μM TCEP, pH 7.4.
